# Purifying Selection on Exonic Splice Enhancers in Intronless Genes

**DOI:** 10.1093/molbev/msw018

**Published:** 2016-01-23

**Authors:** Rosina Savisaar, Laurence D. Hurst

**Affiliations:** ^1^Department of Biology and Biochemistry, The Milner Centre for Evolution, University of Bath, Bath, United Kingdom

**Keywords:** avoidance selection, intronless genes, exonic splice enhancers, SR proteins, splice control

## Abstract

Exonic splice enhancers (ESEs) are short nucleotide motifs, enriched near exon ends, that enhance the recognition of the splice site and thus promote splicing. Are intronless genes under selection to avoid these motifs so as not to attract the splicing machinery to an mRNA that should not be spliced, thereby preventing the production of an aberrant transcript? Consistent with this possibility, we find that ESEs in putative recent retrocopies are at a higher density and evolving faster than those in other intronless genes, suggesting that they are being lost. Moreover, intronless genes are less dense in putative ESEs than intron-containing ones. However, this latter difference is likely due to the skewed base composition of intronless sequences, a skew that is in line with the general GC richness of few exon genes. Indeed, after controlling for such biases, we find that both intronless and intron-containing genes are denser in ESEs than expected by chance. Importantly, nucleotide-controlled analysis of evolutionary rates at synonymous sites in ESEs indicates that the ESEs in intronless genes are under purifying selection in both human and mouse. We conclude that on the loss of introns, some but not all, ESE motifs are lost, the remainder having functions beyond a role in splice promotion. These results have implications for the design of intronless transgenes and for understanding the causes of selection on synonymous sites.

## Introduction

Purifying selection, the purging of deleterious variants from the population, is the most common mode of operation of selection (Kimura 1984). At the molecular level, purifying selection is commonly seen in the maintenance of sequence motifs whose degradation decreases the fitness of the organism. However, there is evidence that selection can also act to avoid a particular sequence motif if its presence in a given context is deleterious. This phenomenon, that we term “avoidance selection,” constitutes another facet of purifying selection and has been documented in a wide variety of biological systems (Sharp 1986; Hahn et al. 2003; Farh et al. 2005; Ackermann and Chao 2006; Cusack et al. 2011; Li et al. 2012; Zur and Tuller 2013; Kupzcok and Bollback 2014). For instance, there is evidence to suggest that the 3′UTRs of mRNA genes that are highly and specifically coexpressed with a microRNA are under selection to avoid complementary sites to the seed region of that microRNA so as to prevent inappropriate downregulation (Farh et al. 2005). Similarly, human exons that are less efficiently monitored by nonsense-mediated decay (NMD), such as final exons, are depleted in codons that are only a single-point mutation away from a stop codon, presumably to enhance robustness to transcriptional or missplicing-induced errors (Cusack et al. 2011).

Here we test a further potential case of avoidance selection. We hypothesized that there could be avoidance of exonic splice enhancers (ESEs) in genes that do not contain introns. ESEs are short nucleotide motifs that are exceedingly common in vertebrate exons, especially near the splice sites (Blencowe 2000; Fairbrother et al. 2004; Wu et al. 2005; Cáceres and Hurst 2013). The classical model for ESE function postulates that these motifs are bound by serine/arginine-rich (SR) proteins, which interact with components of the spliceosome, helping to recruit them to the 5′ and 3′ splice sites and promote the inclusion of the exon in the mature mRNA (Blencowe 2000; Zhou and Fu 2013). SR protein binding can, however, also have splice-inhibitory roles in certain contexts (Kanopka et al. 1996; Erkelenz et al. 2013; Pandit et al. 2013; Bradley et al. 2015).

Importantly, ESEs appear to play a major role in shaping patterns of vertebrate sequence evolution. These motifs are under purifying selection, as evidenced both by their decreased rate of evolution at synonymous and nonsynonymous sites (Parmley et al. 2006, 2007) and by their depletion in single nucleotide polymorphisms (SNPs) (Majewski and Ott 2002; Fairbrother et al. 2004; Carlini and Genut 2006; Cáceres and Hurst 2013). Their abundance at exon ends explains, at least in part, why exon ends tend to be more conserved than exon cores (Majewski and Ott 2002; Fairbrother et al. 2004; Goren et al. 2006; Parmley et al. 2006, 2007). In addition, both codon and amino acid usage are skewed near exon ends in a way that is consistent with an effect of ESEs (Willie and Majewski 2004; Parmley and Hurst 2007; Parmley et al. 2007; Warnecke et al. 2008).

According to our hypothesis, the impact of ESEs on sequence evolution could have an additional dimension—their avoidance in locations where their presence might be deleterious. Specifically, we hypothesized that ESEs might be avoided in intronless genes, as the recruitment of splicing factors to the transcript could theoretically cause the activation of cryptic splice sites and thus lead to inappropriate RNA processing. The deleterious end result could be the production of a malformed mRNA transcript. At the very least, recruitment of SR proteins to sequences not requiring splicing could be considered a waste of resources.

The avoidance hypothesis for ESEs in single-exon genes presumes that their presence in intronless transcripts would be deleterious. This need not, however, be the case. For instance, if an ESE divorced from a splice junction failed to attract its binding partner(s), then the motifs could instead be evolving neutrally. Alternatively, if ESEs serve additional functions beyond a role in coordinating splicing, then selection may in fact act to preserve them (or a subset of them) in intronless genes. Prior evidence showing SR proteins to be involved in various processes all along the gene expression pathway (Huang et al. 2003; Sanford et al. 2004; Zhang and Krainer 2004; Li and Manley 2005; Lin, et al. 2008; Twyffels et al. 2011; Änkö 2014; Howard and Sanford 2015; see Discussion for additional references), as well as work hinting at possible ESE involvement in the nuclear retention of intronless transcripts (Taniguchi et al. 2007) would be consistent with this possibility. Nevertheless, it remains unclear whether or not the benefits of maintaining these additional functions could outweigh any potential deleterious effects of attracting SR proteins to intronless genes.

If avoidance selection truly is acting with regards to ESEs in intronless genes, this will add another layer to our understanding of how these motifs evolve and will further underline the importance of considering avoidance effects in studies of molecular evolution, including selection on synonymous mutations. The study will also contribute to the literature on intronless genes, which stand out in many ways beyond their simple lack of introns (Grzybowska 2012). For example, single-exon genes tend to be more tissue specific, faster evolving, and evolutionarily more recent than multi-exon ones (Shabalina et al. 2010). They are also known to be enriched in particular functional categories, such as signal transduction, and depleted in others, such as catalytic activity (Hill and Sorscher 2006; Louhichi et al. 2011). In addition, a recent study compared nucleosome positioning in intronless and intron-containing genes, notably revealing nucleosome occupancy to be lower in the promoter region yet higher in the gene body for intronless than it is for intron-containing genes (Cheng et al. 2015). Determining whether there is ESE depletion in intronless genes might help us understand these particularities better, given that differences in splice enhancer content will likely translate to differences in terms of the identity of the proteins that contact the RNA and/or the corresponding region of DNA.

The results of this analysis might also have more immediate practical ramifications. In mammalian transgenesis, including gene therapy, it is common to remove all or nearly all introns from the parental gene to generate the transgene. In principle, via modification of synonymous sites, one can remove many ESE motifs from what were exon ends but are in the transgene distanced from any exon boundary. If avoidance selection in intronless genes is witnessed this would appear to be a sensible strategy as it helps prevent incorrect splicing while potentially increasing RNA stability. Conversely, if intronless genes preserve ESEs then such a strategy might be disadvantageous.

## Results

### Intronless Genes Are Less Dense in ESEs than Intron-Containing Genes

**Fig. 1:**
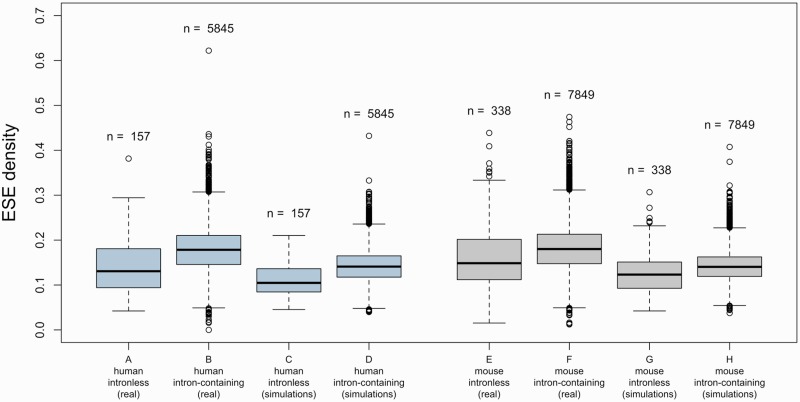
A and B present ESE densities in *Homo* sapiens intronless and intron-containing genes. C and D are the ESE densities obtained for each data point in simulations, averaged over 10,000 iterations. Note that even though ESE density is higher in intron-containing than in intronless genes, the same can be observed in nucleotide-controlled simulations. E–H present the same data for the *Mus musculus* genome.

If intronless genes are under selection to avoid ESEs, they are likely to be less dense in these motifs than intron-containing sequences (although note that this criterion is neither necessary nor sufficient—see below). In order to test this prediction, we compiled a set of 344 intronless human coding sequences (CDSs) and clustered the sequences into paralogous families. Each family was considered as a single data point for the remainder of the analysis (see Materials and Methods). This left a final sample size of 157 data points. We predicted hits to 84 high-confidence ESE motifs in these CDSs, recovering a median ESE density (proportion of nucleotides within a sequence that are part of an ESE) of ≈0.131. We then similarly compiled a set of CDSs from human multi-exon genes (10,337 sequences, 5,845 data points) and scanned it for ESEs. The median ESE density observed across intron-containing sequences was ≈0.178—significantly higher than in intronless genes (A and B in fig. 1; *P* < 2.2 × 10^−^^16^, two-tailed Wilcoxon rank-sum test). Note that we scanned the entirety of the CDS, not just exon ends. This is because exon end has no meaning in the context of intronless genes and thus to perform a relevant comparative analysis we needed to scan all CDS.

**Fig. 2:**
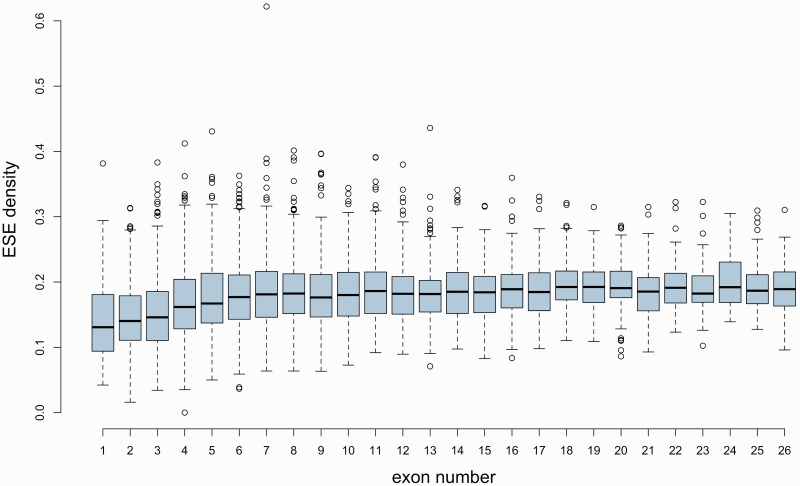
ESE densities of genes with different numbers of exons. Exon number classes with less than 50 observations (exon number above 26) have been removed.

It is suggested that selection for ESEs will depend on the probability of there being downstream decoy splice sites (Wu and Hurst 2015). This being so we might expect low ESE density to more generally be a property of genes with relatively few exons and not of intronless genes alone. We indeed observe a significant positive correlation between the number of exons in a gene and the ESE density of its CDS (fig. 2; ρ ≈ 0.232, *P* < 2.2 × 10^−^^16^, Spearman rank correlation). This correlation is robust to the removal of intronless genes, suggesting that the effect is not driven by the very low ESE density of single-exon sequences (without intronless genes: ρ ** **≈ 0.213, *P* < 2.2 × 10^−^^16^, Spearman rank correlation). Because Spearman’s ρ is not robust to tied values in the data, we repeated the analysis using Goodman and Kruskal’s “gamma” instead. The results were qualitatively similar (with intronless genes: *G* ≈ 0.161, *P* ≈ 9.999 × 10^−^^5^; without intronless genes: *G* ≈ 0.148, *P* ≈ 9.999 × 10^−^^5^). Intronless genes are therefore indeed depleted in ESEs when compared with intron-containing sequences, although there is also a broader effect of exon number that goes beyond a simple dichotomy between single- and multi-exon genes.

### Both Intronless and Intron-Containing Genes Are Denser in ESEs than Expected by Chance

The above analysis, although suggestive, does not, however, directly address the problem of avoidance. For example, the results would also be compatible with a scenario in which the ESEs in intronless genes were evolving neutrally (selection for neither maintenance nor avoidance), while those in intron-containing genes were under selection to be maintained. To test for avoidance, it is necessary to obtain an estimate of the ESE density we would expect to see by chance if no selection was acting on these motifs, given their base composition and the base composition of the sequences. If the actual density was lower, this would constitute evidence for avoidance.

To compute this estimate, we generated 10,000 sets of randomly generated ESE motifs that preserved the dinucleotide frequency of real ESEs and predicted hits to each such set. The average median density obtained in intronless genes in these simulations was only ≈0.107. As this value is smaller than that observed with real ESEs (≈0.131), we can conclude that there is no evidence for avoidance and rather an enrichment (one-tailed *P* ≈ 0.004 from the empirical distribution) in ESEs in intronless genes when compared with random expectations. Intron-containing genes are similarly found to be enriched in ESEs (supplementary fig. S1, Supplementary Material online; one-tailed *P* ≈ 9.999 × 10^−^^5^ from empirical distribution). Similar results were obtained when the random expectation was computed by predicting hits to ESE motifs in 10,000 artificial sets of sequences that had been constructed by shuffling the codons in the real CDSs (supplementary table S2, Supplementary Material online).

**Fig. 3:**
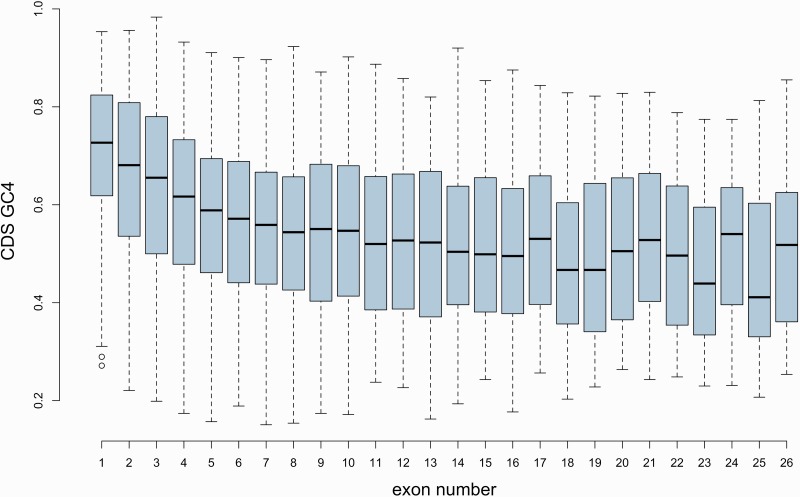
CDS GC_4_ of genes with different numbers of exons.

Importantly, the simulations using artificial hexamers also unveiled that even though the density of real ESEs was higher in intron-containing genes than in intronless ones, the same was true for the simulated motifs, suggesting that the large difference observed with real ESEs might be a consequence of base composition biases (C and D in fig. 1). Namely, single-exon genes have a higher guanine-cytosine (GC) content than do multi-exon genes, and there is a negative correlation between GC content at 4-fold degenerate sites (GC_4_) and exon number (fig. 3; ρ ≈ −0.262, *P* < 2.2 × 10^−^^16^, Spearman rank correlation; *G* ≈ −0.186, *P* ≈ 9.999 × 10^−^^5^, Goodman and Kruskal’s gamma). ESEs, on the other hand, are highly enriched in purines, especially adenines (supplementary table S1, Supplementary Material online). Because of the adenine bias, the set of ESEs used has a slightly higher adenine-thymine (AT) content than GC content. This means that GC-rich sequences (such as single-exon genes) would be predicted to be less dense in these motifs than more AT-rich sequences, even if ESEs are no less functionally relevant to the first set of sequences than they are to the second.

To control for such nucleotide biases in further analysis, the raw density observed for each data point was converted into a normalized density (ND) value by subtracting the mean simulated density for that data point from the actual density and then dividing the difference by the simulated mean ($ND\;=\frac{real\;density\;–\;mean\;of\;simulated\;densities}{mean\;of\;simulated\;densities}$), thereby providing a measure of enrichment over expected. Because the simulations were performed using randomized ESEs that preserved the dinucleotide frequencies of the original set, this normalization amounts to controlling for dinucleotide composition. Median ND was found to be 0.253 for intronless genes and 0.259 for intron-containing ones. There is therefore no significant difference in ND between the two groups (*P* ≈ 0.979, two-tailed Wilcoxon rank-sum test), confirming the intuition that the seeming depletion of ESEs in intronless genes is most likely a consequence of the higher GC content of the sequences. Similarly, the correlation between exon number and ESE density disappears once ND is used instead of raw density (supplementary fig. S2, Supplementary Material online; ρ ≈ 0.019, *P* ≈ 0.158, Spearman rank correlation; *G* ≈ 0.011, *P* ≈ 0.112, Goodman and Kruskal’s gamma), suggesting that the increase in ESE density along with exon number might also largely have been a simple reflection of the decrease in GC content.

There is, however, another factor to consider. Exon number is correlated strongly and positively with the length of the CDS (ρ ≈ 0.691, *P* < 2.2 × 10^−^^16^, Spearman rank correlation; *G* ≈ 0.544, *P* ≈ 9.999 × 10^−^^5^, Goodman and Kruskal’s gamma). This covariate could be confounding any correlation between ND and exon number. In order to better understand the relationships between the variables, we performed a partial Spearman correlation among ND, exon number, and CDS length. We found ND to correlate positively with exon number (ρ ≈ 0.088, *P* ≈ 2.238 × 10^−^^11^) but negatively with CDS length (ρ ≈ −0.108, *P* ≈ 2.475 × 10^−^^16^). This suggests that, as expected from the decoy splice site model, genes with more exons do indeed have a higher ESE density, even after controlling for nucleotide composition effects. This relationship is, however, confounded by a negative correlation between ND and CDS length.

**Fig. 4:**
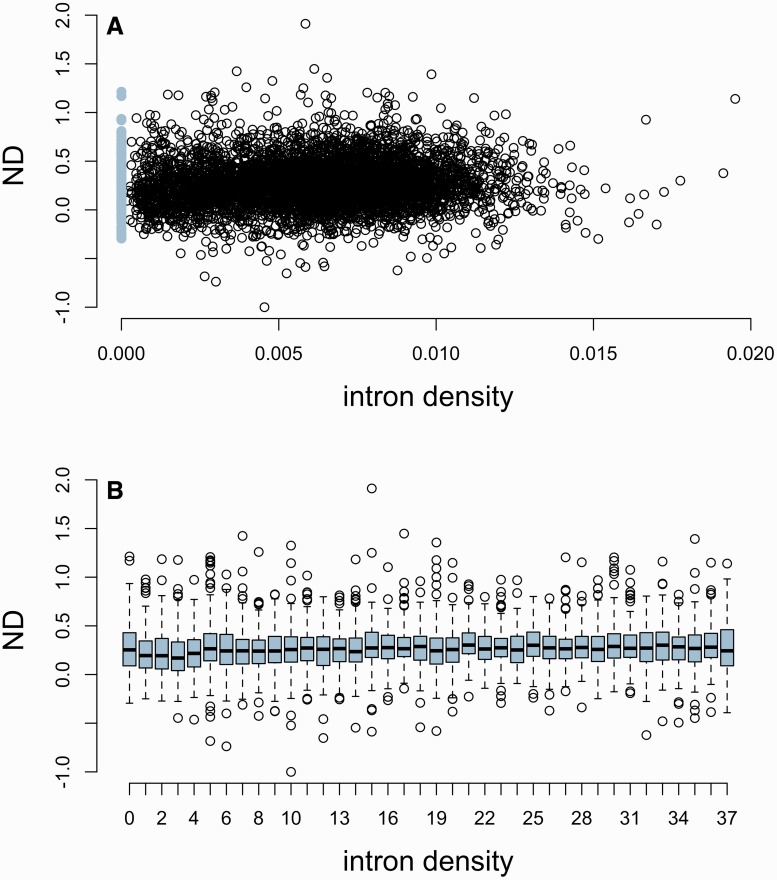
(*A*) Normalized ESE density (ND) in genes with differing intron density. Intronless genes have been highlighted in light blue. (*B*) Normalized ESE density (ND) in genes binned according to intron density. Intronless genes correspond to the first bin. The number of bins was chosen to obtain roughly the same number of genes in each intron-containing bin as in the intronless one.

We therefore chose to consider not exon number but intron density (number of introns per base pair of CDS), a measure that combines the parameters of exon number and CDS length, and has been shown previously to correlate with ESE density (Wu and Hurst 2015). Single-exon genes can then be redefined as the set of genes having an intron density of 0. We found a weak but significant correlation between intron density and ND (ρ ≈ 0.088, *P* ≈ 3.069 × 10^−^^11^, Spearman rank correlation). Most importantly, however, figure 4 shows that ND is not particularly low in intronless genes specifically and, if anything, is even slightly higher than in other genes with low intron density.

We conclude that ESE density is indeed higher in genes with more exons but when compared with random expectations, the motifs are enriched, not avoided, in intronless genes. The latter result also holds in mouse (*Mus musculus*) intronless genes, which are similarly enriched in ESE hexamers (intronless genes: raw median density ≈ 0.149, median ND ≈ 0.279, enrichment *P* ≈ 0.0003; intron-containing genes: raw median density ≈ 0.180, median ND ≈ 0.279, enrichment *P* ≈ 9.999 × 10^−^^5^; see fig. 1 and supplementary fig. S1, Supplementary Material online).

### Evidence that ESEs in Intronless Genes Are under Purifying Selection

We propose two possible explanations for the finding that intronless genes are enriched in splice enhancer motifs, relative to nucleotide-controlled null, despite the fact that the corresponding transcripts presumably do not undergo splicing. The first would be that ESEs have additional, splicing-independent roles in the cell, an explanation that is consistent with a body of experimental work on SR protein function (see Discussion for details). The second explanation, more prosaic, would be that the enrichment merely reflects the recent origin of intronless genes from parental intron-containing sequences and a time lag in the loss of the motifs. In the first case, the ESEs in intronless genes would be functional and we should be able to detect signs of purifying selection acting on them. In the latter case, however, the motifs would be expected to be evolving at the rate expected by chance or even faster if the sequences were under selection to lose such inappropriate splice enhancers.

In order to test these predictions, human ESE regions were aligned to homologous regions in rhesus macaque (*Macaca mulatta*) and the rate of evolution at synonymous sites (*d_S_*) was calculated. The same protocol was then repeated with the ESEs in each of the randomized sets, creating an empirical distribution. The results show the synonymous sites in ESEs in intronless genes to be evolving ≈20.8% more slowly than expected from dinucleotide-controlled simulations (observed *d_S_* ≈ 0.051, expected *d_S_* ≈ 0.065, *P* ≈ 2.000 × 10^−^^4^, one-tailed from empirical distribution). The results are comparable for intron-containing genes (observed *d_S_* ≈ 0.056, expected *d_S_* ≈ 0.062, *P* ≈ 0.001). A similar analysis was conducted on ESE regions in mouse CDSs, with rat orthologs used to calculate *d_S_*. ESEs were once again found to evolve significantly slower than control regions, in both single- and multi-exon genes (single exon: observed *d_S_* ≈ 0.151, expected *d_S_* ≈ 0.161, *P* ≈  0.043; multi exon: observed *d_S_* ≈ 0.153, expected *d_S_* ≈ 0.167, *P* ≈ 0.002).

In addition, SNP density at 4-fold degenerate sites within human ESE regions was calculated and, in both intronless and intron-containing genes, was found to be marginally lower than expected (table 1). However, in both cases, the decrease was nonsignificant. As we considered intron-containing genes as a positive control, we concluded that the test most likely did not have sufficient power on our data set.

**Table 1.** SNP Density in Intronless and Intron-Containing Genes.

	Intronless Genes	Intron-Containing Genes
SNP density in real ESEs	≈0.044	≈0.047
Mean SNP density in simulated ESEs	≈0.046	≈0.051
Normalized SNP density $\left(\frac{real\;–\;simulated}{simulated}\right)$	≈−0.028	≈−0.078
One-tailed *P* value from empirical distribution	≈0.395	≈0.106
Sample size	157 data points (344 genes)	5,845 data points (10,337 genes)

Purifying selection is also expected to leave a relative excess of low frequency variants in polymorphism data (Kimura 1984). We therefore calculated the fraction of segregating sites within ESEs that showed a minor allele frequency (MAF) of less than 1/2,000, an arbitrary threshold that we set for classifying a variant as rare. We then performed the same analysis using 10,000 simulated ESEs and used the empirical distribution for the fraction of low MAF sites thus obtained to determine significance. However, because we included a random member from each paralogous family, our calculations had a stochastic component and we found considerable fluctuations in the results depending on which exact sequences were used. We therefore repeated the analysis ten times and report the median fraction of low MAF sites and the median one-tailed empirical *P* value as our final statistics. In this manner, we found a median fraction of segregating sites with a low MAF of ≈0.295 (range from ≈0.274 to ≈0.313) in ESEs in single-exon genes and of ≈0.380 (range from ≈0.377 to ≈0.385) in multi-exon genes, the first fraction being significantly and the second nearly significantly higher than expected from simulations (median one-tailed *P* values ≈0.017 and ≈0.053, respectively). This suggests that the excess in low frequency variants is indeed greater for intronless gene ESEs than it is for surrounding dinucleotide-matched CDS, a result that is expected if the regions are under selection not only because of their role in specifying the amino acid sequence but also for noncoding functions.

The decreased rate of evolution at synonymous sites, as well as the excess of rare variants in polymorphism data, lead us to conclude that there is evidence for purifying selection acting on the ESEs in single-exon genes and that at least some of them are therefore likely to be functional.

### ESEs Are Both Slightly More Frequent and Faster Evolving in Putative Recent Retrocopies than in Other Intronless Genes

As discussed above, the hypothesis that the ESE enrichment observed in single-exon sequences was purely due to nonfunctional motifs inherited from intron-containing parent genes would predict the ESEs to be evolving neutrally. The signs of purifying selection that we uncovered in intronless gene ESEs therefore constitute strong evidence against it. However, a weaker version of the hypothesis could still be correct: Even though some of the ESEs in intronless genes would be functional, inherited nonfunctional motifs could still be present and inflate our estimate of ND.

If this revised version of the second hypothesis were true then those genes that result from recent retroposition events should stand out from other, more ancient intronless genes. Concretely, we would expect such recent retrocopies to have higher ND than other intronless genes because the ESEs that they inherited from their intron-containing parents would have had less time to disappear through drift (i.e., there would be evolutionary lag). Their removal from the set of intronless CDS should therefore cause the overall ND to drop. Second, we would expect the ESEs in such sequences to be evolving faster than those in other intronless genes as a greater proportion of the motifs would be nonfunctional.

In order to test these predictions, we screened our data set of intronless genes for overlaps with regions annotated as retrocopies in the UCSC Genome Browser RetroGenes V9 track. 82 out of the 344 intronless genes were seen to overlap 50% or more with a retrocopy region (“broad retrocopies set”). Twenty-one of these could be successfully aligned to an intron-containing parent gene (“strict retrocopies set”; see Materials and Methods for details). Supplementary text S1 and figure S4, Supplementary Material online, characterize the strict retrocopies set with regards to how their usage of ESEs compares with that of their parents, but because of the very small sample size, these data are of descriptive value only and will not be reproduced in the main text.

We then investigated the effect of removing these retrocopies from the data set on the overall ND. When broad set retrocopies were excluded, the estimator decreased somewhat, from ≈0.253 to ≈0.233. The effect on the enrichment *P* value was greater, with the probability of observing an ESE density as high or higher by chance rising from ≈0.004 to ≈0.015 (table 2).

**Table 2.** Raw and Normalized ESE Density (ND) in Putative Retrocopies, in Other Intronless Sequences, and in the Full Set of Intronless Genes.

	Full Data Set	Without Broad Set Retrocopies	Broad Set Retrocopies Only
Median raw ESE density	≈0.131	≈0.121	≈0.156
Median ND	≈0.253	≈0.233	≈0.275
*P* value for enrichment over expected^a^	≈0.004	≈0.015	≈2.000 × 10^−^^4^
Sample size^b^	157 data points (344 genes)	122 data points (262 genes)	50 data points (82 genes)

^a^Here, the *P* value is the probability that an ESE density this high or higher could have been obtained by chance given the nucleotide composition of the sequences. It was computed separately for each set and does not pertain to a comparison between sets.

^b^The sample size differs from the number of genes because each data point corresponds to a paralogous family rather than to a single gene (see Materials and Methods). The clustering into families was performed independently for each data set.

These findings are difficult to interpret, however, as any change to the makeup and size of the sample is likely to have some effect on the estimators. We therefore performed a simulation to determine whether the effect that excluding the retrocopies had on ND and on the associated *P* value was in accord with what would be expected after removing a subset of this size. Over 1,000 iterations, we randomly removed 82 genes from the intronless data set and counted how many times this caused a decrease in ND or an increase in *P* value as great as or greater than that observed when removing the broad retrocopies set. We found that for most simulants, the changes to the estimators were not as great as those observed when true retrocopies were removed (*P* ≈ 0.022 for *P* value, ≈ 0.030 for ND). Recent retrocopies therefore do contribute disproportionately to the overall ND observed. Importantly, however, ESE density remains significantly higher than expected by chance even after the retrocopies are removed (table 2 and supplementary fig. S3*a* and *b*, Supplementary Material online).

The second prediction was that the ESEs in putative recent retrocopies should be evolving faster than those in other intronless genes. This is indeed what we observed after calculating the *d_S_* rate and SNP density at 4-fold degenerate sites in ESEs in both the broad retrocopies set and in all remaining intronless sequences. The *d_S_* rate of ESEs in the broad retrocopies set was still lower than expected from simulations but not significantly so (supplementary table S4 and fig. S3*c* and *d*, Supplementary Material online), whereas the SNP density was actually ≈10.2% higher than expected (supplementary table S5 and fig. S3*e* and *f*, Supplementary Material online). The latter result might shed light on why the depletion in SNPs observed for the ESEs in the full set of intronless genes (≈2.8%) was so marginal—it could be that the presence of fast-evolving ESEs in recent retrocopies increased the SNP density estimate for the whole set. When broad set retrocopies were removed, the extent of the SNP depletion in intronless gene ESEs was ≈14.2%, a much greater effect size than for the full set although still not quite significant (*P* ≈ 0.092, one-tailed from empirical simulation).

Similarly to the test carried out above for ESE density, we wanted to estimate the probability that the decrease in SNP density observed when putative retrocopies were removed was a mere side effect of the change in sample size. We therefore repeated the analysis 1,000 times, excluding a random set of 82 genes each time, and noted how often this resulted in a decrease in SNP density or an increase in *P* value as great as or greater than that observed when removing real retrocopies. The results indicated that the effect was unlikely to be an artifact of the reduction in sample size (*P* ≈ 0.037 for *P* value, ≈ 0.027 for normalized SNP density), suggesting that the ESEs in putative retrocopies were indeed a particularly polymorphism-rich subset of all ESEs in intronless genes.

We infer from these results that two classes of motifs likely contribute to the ESE enrichment observed in intronless genes. The first is composed of ESEs that are probably functional and are selectively maintained because they carry out splicing-independent roles. The second, however, corresponds to motifs that are mere functionless ghosts of the intron-containing past and are destined to eventually disappear, whether it be through drift or perhaps through avoidance selection against the presence of such inappropriate splice enhancers.

### Certain ESEs Are Overrepresented in Genes with Longer Introns, Others in Genes with Shorter Introns

If ESEs have additional, splicing-independent roles, as the results above would suggest, then this could mean that there are functional differences between ESEs, with some largely specialized to splicing and others more likely to carry out other functions. This would be expected as preferred binding sites vary between SR proteins (Liu et al. 1998; Änkö 2014) and there is evidence to suggest that these proteins are functionally heterogeneous. For instance, some have been found to shuttle between the nucleus and the cytoplasm, whereas others do not seem to do so (Cáceres et al. 1998; Sapra et al. 2009). Similarly, ESEs differ in their capacity to enhance translation depending on the SR proteins that bind them (Sanford et al. 2004). We further speculated that if different genes, depending on their architecture, were more or less likely to rely on ESEs for splicing (rather than for other functions), this should lead to variation between CDSs in the frequencies with which different ESE motifs were used. This, in turn, could allow us to isolate functional classes of ESEs.

Concretely, we focused on intron size as one particular aspect of gene architecture. Several previous studies have found ESE density to correlate positively with the size of the flanking intron (Dewey et al. 2006; Warnecke et al. 2008; Cáceres and Hurst 2013; Schüler et al. 2014; Wu and Hurst 2015). This likely reflects the importance of ESEs for splicing as evidence suggests the process to be more error-prone (and, consequently, perhaps more likely to need reinforcement through ESEs) when introns are larger (Fox-Walsh et al. 2005). We hypothesized that this positive correlation between motif frequency and intron size should be weaker for ESEs that have important splicing-independent roles because their distribution should be less predominantly determined by splicing-related constraints. A prediction of the hypothesis is that these ESEs, as a reflection of their presumed multifunctionality, should also be particularly common in intronless genes when compared with other putative splice hexamers.

**Fig. 5:**
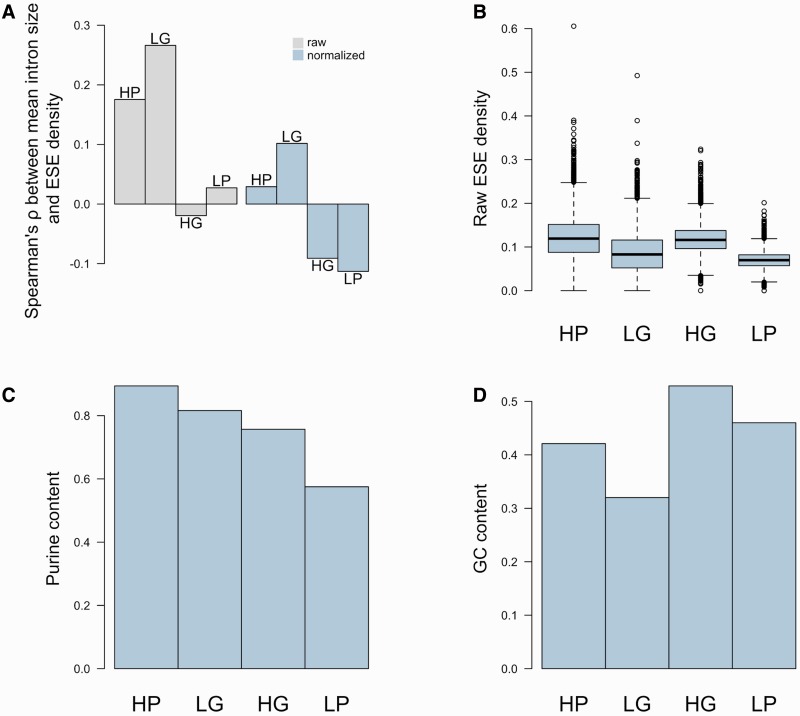
(*A*) Spearman rank correlation coefficient between raw ESE density (grey bars) or normalized ESE density (ND) (blue bars), and mean intron size calculated using different sets of ESEs. (*B*) Raw densities of different subsets of ESEs. (*C*) Purine content in the different sets of ESEs. (*D*) GC content in the different sets of ESEs.

The set of 84 ESE motifs was divided in two along two different partitions, creating, on the one hand, a high purine content set (55 motifs) and a low purine content set (29 motifs), and on the other, a high GC content set (46 motifs) and a low GC content set (38 motifs) (supplementary spreadsheet S2, Supplementary Material online). Hits were then predicted for all four sets and a partial Spearman correlation performed between ESE density (raw or normalized) and mean intron size. We found that only high purine and low GC sets showed the expected positive correlation between mean intron size and ND, while the correlation was negative for the low purine and high GC sets (fig. 5 and supplementary spreadsheet S3, Supplementary Material online).

It is unclear whether this trend more closely follows the purine or GC content of the motifs but, importantly, it is not easily explained by their raw median density (used as a proxy for the amount of information available), suggesting that the pattern observed reflects actual distribution differences between motifs with differing base composition and is not an artifact of a dearth of sites and thus statistical power for some subsets (fig. 5; see supplementary fig. S5, Supplementary Material online, for the results, near identical, that are obtained after the removal of putative recent retrocopies). In this context, normalization for dinucleotide content is crucial: There is a negative correlation between GC_4_ and mean intron size (ρ ≈ −0.280, *P* < 2.2 × 10^−^^16^, Spearman rank correlation), a relationship that is likely a reflection of the tendency for exons flanked by large introns to have a higher AT content (Duret et al. 1995; Hurst et al. 1999; Carels and Bernardi 2000; Amit et al. 2012). This latter trend probably inflates any estimate of the positive correlation between high ESE density and large flanking intron size and as a result the fact that some motifs seem to be enriched near smaller introns only becomes apparent after normalization (supplementary spreadsheet S3, Supplementary Material online).

Such simple distinctions based on the nucleotide composition of the motifs are useful for establishing that there indeed is a link between the structure of a gene and the enrichment patterns of different ESEs. However, the insights that they can provide are superficial and might overlook more subtle differences in distribution between particular ESEs. We therefore sought to examine differences in usage between particular splice enhancer hexamers.

In order to minimize contamination from nonfunctional motifs inherited from intron-containing parents, we first removed broad set retrocopies from the sample of intronless genes. We then constructed a matrix where element *i*, *j* corresponded to the number of bases in gene *i* that overlapped with instances of ESE *j* (averaging the counts from paralogous families). As the next step, we binned our data points based on mean intron size, with intronless genes (mean intron size 0) forming the first bin and intron-containing genes divided into approximately equal groups along every 1/48^th^ quantile, resulting in a total of 49 intron size bins. This bin number was chosen because for the intron-containing genes, it generated bins that contained an average of ≈121.771 data points, a close match to the size of our intronless sample (*n* = 122). We then summed the counts obtained for all the data points in a particular bin and divided the result by the sum of the lengths of the corresponding CDSs (once again averaging over paralogous families), providing a single estimate for the density of each motif in each intron size bin. We organized these estimates in a 49 × 84 matrix with intron size bins in the rows and ESEs in the columns (see supplementary spreadsheet S4, Supplementary Material online, that also has details on the bin boundary values).

For each ESE, we then created a control set made up of the 60 hexamers that could be generated by permuting the dinucleotides in the original motifs. This allowed for 60 simulations where we predicted hits to a different set of 84 simulated motifs each time, each simulated hexamer corresponding to a particular ESE in the actual set. For each simulation, we could then construct a 49 × 84 matrix, similarly to what was described above for real ESEs. Finally, we calculated the median obtained across all simulants of a particular ESE motif in a given intron size bin and used it to produce an ND measure for that cell of the matrix by subtracting the simulated median from the real ESE density observed and dividing the difference by the simulated median. This resulted in a matrix that had been normalized for background nucleotide composition (supplementary spreadsheet S5, Supplementary Material online). This is different from the method used above when normalizing overall ESE density—earlier, dinucleotides were pooled across all ESEs and simulants constructed by sampling from that pool. In the analysis performed in the current section, however, each simulated hexamer corresponded to a specific ESE and was constructed from the dinucleotides in that ESE alone. Note that the relative frequencies of different ESEs, normalized in this way, are reproducible between species—there is a strong correlation between the ND of the various hexamers in human intronless genes and in mouse intronless genes (ρ ≈ 0.867, *P* < 2.2 × 10^−^^16^; see also supplementary spreadsheets S6 and S7, Supplementary Material online).

**Fig. 6:**
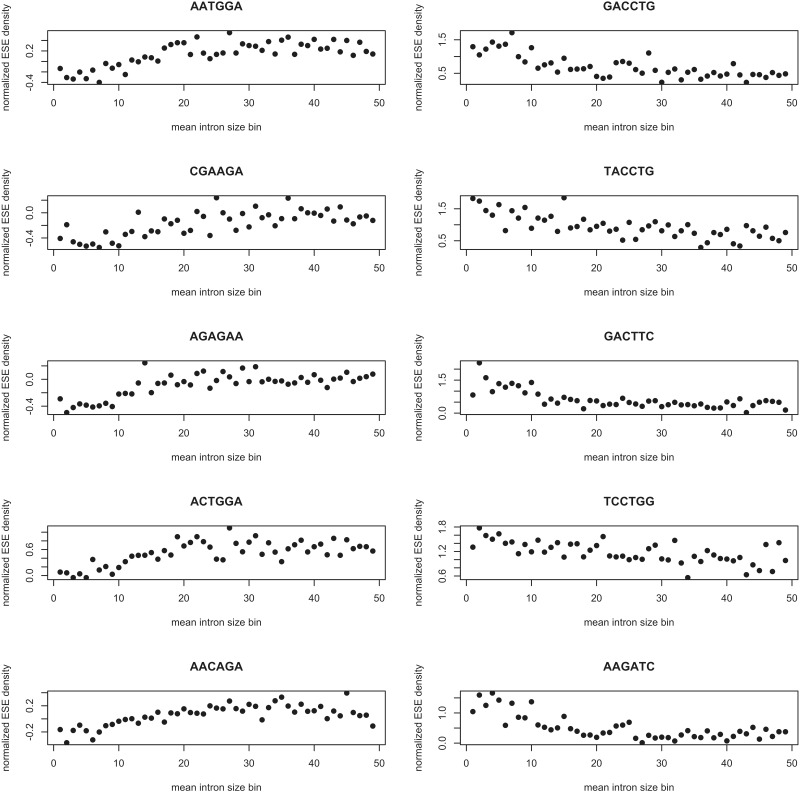
ND of various ESEs in different mean intron size bins. The first dot (bin 0) represents intronless genes. The column on the left corresponds to the five motifs that give rise to the strongest positive correlation, while the plots on the right depict the ND of the five motifs presenting the strongest negative correlation.

We next proceeded to calculate the correlation between each column of the matrix and intron size bin indices (a vector of integers from 0 to 48), and Holm-corrected the resulting *P* values for multiple comparisons. After the correction, the ND of 36 of the 84 ESEs was found to be significantly correlated with intron size bin indices, with 12 motifs showing a positive (preference for larger introns) and 24 a negative (preference for smaller introns) correlation (fig. 6 and supplementary spreadsheet S8, Supplementary Material online). That the normalized distribution of both sets of motifs correlates with an aspect of exon–intron architecture but with the inverse sign might suggest that the hexamers play slightly different roles in splicing. Importantly, it does not seem that the negative correlation with mean intron size simply reflects a preference for shorter genes, as in a partial Spearman correlation among the ND of low purine motifs, mean intron size, and genomic length (length of the sequence from the start to the stop, including intervening introns), the correlation between ND and mean intron size is significant (ρ ≈ −0.071, *P* ≈ 2.898 × 10^−^^8^) while that between ND and genomic length is not (ρ ≈ −0.014, *P* ≈ 0.285).

In this light, it is interesting to note that the two sets of ESEs also distribute differently along the exon. ESEs are known to be more potent in their splice enhancer function when they appear in the proximity of the splice junction (Graveley et al. 1998) and, accordingly, their frequency increases toward the exon end (Fairbrother et al. 2004; Wu et al. 2005; Dewey et al. 2006; Cáceres and Hurst 2013), a trend that is also apparent in our data (supplementary fig. S6, Supplementary Material online). However, when we calculated the ND of either set of hexamers in the upstream-most 69 bp and in the 69 bp in the middle of 4,613 exons, we found that this tendency was stronger for the ESEs that are enriched when introns are larger. These motifs presented a flank ND that was ≈2.909-fold greater than the core ND, while for the other set of hexamers the corresponding ratio was ≈1.018. The difference between the two ratios was nearly significant (one-tailed *P* ≈ 0.055 from empirical distribution) and might suggest that the motifs enriched near small introns either have more predominant splicing-independent functions or, alternatively, are involved in splicing in a way that is less constrained by distance to the splice site (supplementary fig. S7, Supplementary Material online).

In conclusion, although some ESEs are indeed enriched more in genes with larger introns, as expected from the previous literature, an even larger subset shows the opposite pattern and is at a higher nucleotide-controlled density when introns are smaller. We suggest that the positive correlation between ESE density and flanking intron size that has been reported in previous studies (Warnecke et al. 2008; Cáceres and Hurst 2013; Schüler et al. 2014; Wu and Hurst 2015) might have been inflated by a failure to account for the lower GC content of exons flanked by large introns (Duret et al. 1995; Hurst et al. 1999; Carels and Bernardi 2000; Amit et al. 2012) (note that Dewey et al. 2006, who do control for this confound, only obtain a weak positive correlation for short introns and no effect otherwise).

### The ESEs that Are Enriched When Introns Are Smaller Are Particularly Likely to be Multifunctional

**Fig. 7:**
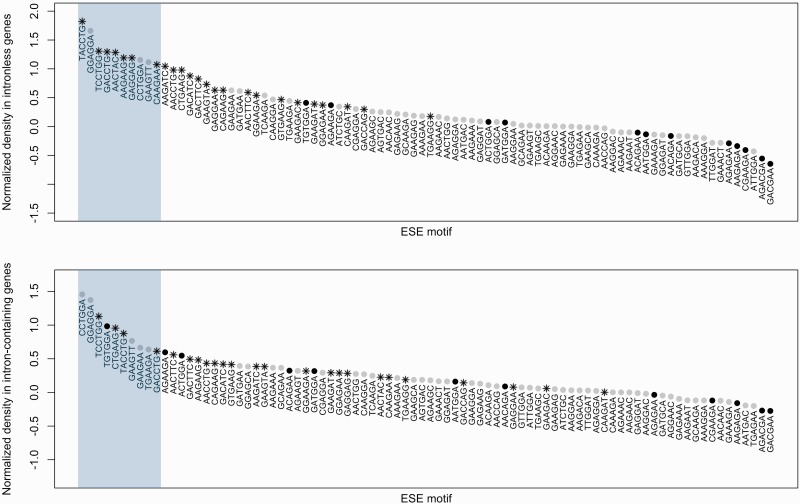
The top panel shows the ND of different ESEs in intronless genes. The blue rectangle highlights the ten most frequent motifs. The shape and color of the dot give information on the correlation between the ND of that motif and mean intron size bin indices. Grey circle: no significant correlation; black circle: significant positive correlation; black star: significant negative correlation. The bottom panel is identical, except that instead of the ND in intronless genes, it is the median ND across intron-containing bins that is plotted.

We hypothesized above that the ESEs that were most likely to be multifunctional and therefore most strongly enriched in intronless genes should be those for which the tendency to occur near larger introns was the weakest. We therefore examined the ten hexamers that were most strongly enriched in intronless genes (fig. 7). We found indeed that none of them presented an ND that was significantly and positively correlated with mean intron size, as per our hypothesis. Importantly, however, 7 of the 10 were among the 24 ESEs that were enriched significantly more in genes with smaller introns, a greater proportion than expected by chance (*P* ≈ 0.008; binomial test of 7 successes out of 10 trials with a probability of success of $\frac{24}{84}$).

More generally, there was a strong negative correlation between a motif’s ND in intronless genes and the coefficient of the Spearman rank correlation between its ND in different mean intron size bins and the intron size bin indices (ρ ≈ −0.758, *P* ≈ 2.2 × 10^−^^16^), indicating that the stronger a motif’s tendency to prefer genes with smaller mean intron size, the higher its frequency in intronless genes. The same was true, although less prominently, when the median ND across all intron-containing bins was considered instead of ND in intronless genes, suggesting that the motifs preferred with smaller introns were also more frequent in intron-containing genes (ρ ≈ −0.389, *P* ≈ 2.851 × 10^−^^4^). Consequently, when we calculated the correlation between ND and mean intron size for all 84 ESEs, the overall correlation coefficient came out as negative (ρ ≈ −0.059, *P* ≈ 4.509 × 10^−^^6^; Spearman rank correlation).

Taken together, these data could indicate that the ESEs that are overrepresented in genes with smaller introns are particularly good candidates for motifs with splicing-independent functions, while those that prefer genes with larger introns could be more specialized to splicing (fig. 6 and supplementary spreadsheet S8, Supplementary Material online). The hypothesis of a stronger specialization to splicing for some motifs than for others leads to a prediction regarding the distribution of the hexamers in putative recent retrocopies versus in other intronless genes. Namely, the motifs that we suspect to be more specialized to splice site recognition should be more common in the probable recent retrocopies than in other single-exon sequences while this trend should be weaker for the presumed multifunctional ESEs. This is so because ancestrally intronless genes and ancient retrocopies should be enriched first and foremost in functional hexamers while recent retrocopies have a higher probability of still being enriched also in splice motifs that were needed in the intron-containing parent but no longer are in the modern intronless CDS.

In order to test this prediction, we calculated the density of the 24 ESEs that were significantly more enriched with decreasing intron size (the putative multitaskers) and of the 12 motifs that showed the opposite pattern (the presumed splice specialists) in both broad set retrocopies and in all other intronless sequences. The putative splice-specialist hexamers indeed showed a higher ND value in probable recent retrocopies (ND ≈ 0.057) than in other intronless genes (ND ≈ −0.110) but this difference was not significant (*P* ≈ 0.358, Mann–Whitney *U* test comparing the ND values; table 3). Hypothesized multifunctional ESEs were also slightly more enriched in broad set retrocopies than in other intronless genes but the difference was much smaller and even less significant (*P* ≈ 0.923, determined as above; table 3). The direction of the effects is therefore consistent with our predictions but because of their nonsignificance, the results do not provide evidence for our hypothesis.

**Table 3.** Normalized ESE Density (ND) of Two Subsets of ESEs in Broad Set Retrocopies and in Other Intronless Genes.

	ESEs Enriched More in Genes with Smaller Introns	ESEs Enriched More in Genes with Larger Introns
ND in broad set retrocopies (one-tailed enrichment *P* value^a^ from simulations)	≈0.936 (*P* ≈ 9.999 × 10^−^^5^)	≈0.057 (*P* ≈ 0.392)
ND in other intronless genes (one-tailed enrichment *P* value^a^ from simulations)	≈0.895 (*P* ≈ 9.999 × 10^−^^5^)	≈−0.110 (*P* ≈ 0.408)

^a^The *p* values correspond to the empirically derived probability that an ESE density this high or higher could have been obtained by chance and does not pertain to any comparisons between subsets of ESEs.

In conclusion, we found that the ESEs that are more strongly enriched when introns are small tend to have a higher ND than those that show the opposite tendency. Because this pattern also holds for intronless genes, we speculate that these ESEs are likely to have important splicing-independent roles.

## Discussion

### ESEs Are Both Enriched and Conserved in Intronless Genes, Suggesting that at least Some Have Roles in Processes Other than Splicing

The aim of this study was to test the hypothesis that ESEs are avoided in intronless genes, as we speculated that an abundance of such motifs could lead to a potentially deleterious recruitment of the splicing machinery to a transcript that should not be spliced. Although raw ESE density is indeed low in intronless genes (as in other genes with few exons), we found intronless genes nevertheless to be “denser” in ESEs than expected by chance given their nucleotide composition.

 We considered the possibility that the reason that ESEs were at a higher density than expected in intronless genes was that recent retrocopies in the set had inherited motifs from their intron-containing parent genes. According to this hypothesis, the ESEs in single-exon CDSs would be nonfunctional in their current context and would be expected to disappear soon through drift. This we find explains some, but not all of the trends. We found that retrocopies were indeed slightly denser in ESEs than other intronless sequences and contributed disproportionately to the overall enrichment. Intriguingly, we also found the ESEs in putative recent retrocopies to be evolving faster than those in other intronless sequences, suggesting that a retroposition event is followed by a period of ESE loss (it is unclear whether simply through drift or whether avoidance selection might be acting), as motifs that were important for splicing in the intron-containing parent are no longer needed in the retrocopy. Importantly, however, we find the rate of evolution at synonymous sites in intronless gene ESEs to be significantly reduced when compared with random expectations, suggesting that at least a subset of the hexamers is under purifying selection and thus functional. The evolutionary lag model cannot account for this finding and therefore only partially explains our observations. It should be noted here that even though our study was primarily conducted on the human genome, we found ESEs to be both enriched and conserved also in mouse intronless genes, providing further evidence that the patterns we report are biologically meaningful.

Our data are thus consistent with some ESEs having functions beyond splice promotion. Another explanation for the ESE enrichment in intronless CDS could, however, be that many of the genes that we consider here as single exon perhaps really do undergo splicing and have simply been mis-annotated. A recent study (Marquez et al. 2015) found evidence for splicing in several human genes annotated as intronless. However, only one of these (*ENSG00000165572*) appears in our data set of intronless sequences. Moreover, as it is part of a two-gene family of paralogs, it only contributes 0.5 data points to the ESE enrichment analysis. Because of the marginality of the overlap, it seems unlikely that such annotation errors could explain the entirety of our observations, although the list presented in Marquez et al. (2015) is probably not exhaustive and thus the explanation cannot be ruled out completely.

We infer that at least some ESEs are likely to have additional functions unrelated to splice enhancement, explaining why intronless genes would show both an enrichment in and conservation of splice enhancer hexamers. It is important to emphasize here that when we claim ESEs to be functional in intronless genes, we use the term “functional” in the evolutionary sense of “relevant to organismal fitness” rather than to merely report biochemical activity (Graur et al. 2013). ESE involvement in splicing-independent processes (e.g., nuclear retention [Taniguchi et al. 2007] or translation [Sanford et al. 2004]) has previously been observed in in vitro assays but to our knowledge there has been no evolutionary investigation into the matter. It has therefore remained unclear whether any such additional functions are merely secondary and anecdotal in comparison with a primary role in splicing or whether they contribute significantly to the overall impact of ESEs on genome evolution. The finding that ESEs are under purifying selection in intronless genes suggests that whatever be the relevant splice promotion independent functions, they play an important role in shaping the selection pressures acting on these motifs and therefore need to be taken into consideration when studying ESEs.

Our conclusions accord with a body of experimental work showing SR proteins to have various splicing-independent roles in the cell (Zhong et al. 2009; Twyffels et al. 2011). The processes in which these proteins appear to be involved include transcriptional elongation (Lin et al. 2008; Ji, et al. 2013; Paz et al. 2014), promoting genome stability (Li and Manley 2005; Xiao et al. 2007; Tuduri et al. 2009), nucleocytoplasmic export of mRNAs (Huang and Steitz 2001; Huang et al. 2003; Lai and Tarn 2004), translation (Sanford et al. 2004; Bedard et al. 2007; Michlewski et al. 2008; Sato et al. 2008; Maslon et al. 2014), regulation of mRNA stability (Lemaire et al. 2002), microRNA processing (Wu et al. 2010), and NMD of mRNAs (Zhang and Krainer 2004; Sato et al. 2008).

As a final caveat we note that we have assumed throughout the article that ESE functionality in intronless genes would imply that the motifs have splicing-independent roles. Strictly speaking, however, we can only infer that they have functions independent of splice enhancement. In other words, the ESEs in intronless genes could be acting as splicing suppressors to counteract any inappropriate positive splicing signals (Kanopka et al. 1996; Pandit et al. 2013; Bradley et al. 2015). Most likely, the ESEs in intronless genes act both in splicing-independent and in splice repressor capacities. Our results do not enable us to determine the relative importance of these two sets of roles and we will continue with the hypothesis that at least some of the ESE enrichment observed in intronless genes reflects wholly splicing-independent functionality—a likely assumption given the extent of SR protein involvement in the various stages of gene expression, reviewed in more detail below.

### ESE Usage Covaries with Gene Structure, Enabling Us to Identify a Set of Motifs that Are Particularly Likely to Have Splicing-Independent Functions

We hypothesized that different SR proteins, and thus potentially also different ESEs, could be more or less specialized to splicing-related versus splicing-independent roles. If in addition sequences with differing architectures relied on ESEs more or less in these different capacities, this should result in gene architecture covarying with the density of particular ESE motifs. We therefore speculated that by examining the relationship between gene structure and ESE usage, we could potentially identify functional subgroups of ESEs.

Concretely, we sought to determine whether there was a relationship between the mean intron size of a gene and the densities of various ESEs, controlling for base composition biases. We chose to examine this parameter because previous work had found ESEs to be more common near larger introns (Dewey et al. 2006; Warnecke et al. 2008; Cáceres and Hurst 2013; Schüler et al. 2014; Wu and Hurst 2015). To our surprise, we discovered that a large subset of the ESEs showed the opposite trend, that is to say, they were more enriched in genes with smaller mean intron size. Moreover, after normalization for nucleotide composition, these motifs were more frequent overall than those that showed the expected tendency to occur in genes with larger introns and were also among the most common motifs in intronless sequences. We concluded that these hexamers were good candidates for ESEs with additional, splicing-independent roles.

It should also be noted that it is probable that our study underestimates the proportion of multifunctional motifs. This is because the computational approach used to derive the set of ESEs was largely based on preconceptions on how the motifs might distribute (Cáceres and Hurst 2013). These, however, were mainly the result of splicing-related considerations. For instance, ESEs were speculated to be more frequent near weaker splice sites. ESE hexamers less implicated in splicing might not be as likely to follow such biases in distribution and could therefore easily escape detection.

Intriguingly, a previous paper (Pozzoli et al. 2004) also compared ESE density in intronless and intron-containing genes, and found it to be higher in the former. This differs from our results, seeing that we found raw ESE density to be significantly greater in multi-exon than in single-exon genes. However, in Pozzoli et al. (2004), ESEs were predicted using SELEX-derived consensus matrices to four SR proteins (Cartegni 2003), an approach quite different from that used to construct the consensus INT3 set used for the work reported here (Cáceres and Hurst 2013). A possible explanation for the discrepancy is that the previous study might have predominantly detected the kind of hexamers that we found to be preferred in genes with smaller mean intron size. It should also be pointed out that the previous authors compared intronless CDSs with intron-containing exons, whereas we compared full intronless CDSs with full intron-containing CDSs.

There are also several possible explanations as to why we uncover an overall negative correlation between nucleotide-controlled ESE density and mean intron size, whereas a positive correlation would have been expected based on the previous literature. Wu and Hurst (2015) and Cáceres and Hurst (2013) predicted hits to the same set of ESEs as was used in this study but did not control for nucleotide composition (when raw rather than normalized ESE density is considered, we also retrieve an overall positive correlation between ESE density and mean intron size; ρ ≈ 0.153, *P* < 2.2 × 10^−^^16^, Spearman rank correlation). Dewey et al. (2006) did control for base composition biases but used a different method of normalization to do so and also employed a different set of ESEs. A final methodological difference is that although previous studies predicted ESEs at exon ends and correlated their density with the size of the flanking intron, in this study we analyzed whole CDSs and used the mean intron size of the gene as our statistic. It should be pointed out, however, that there do not seem to be major differences between motif preferences in whole multi-exon CDSs and at 5′ ends of exons (compare fig. 7 and supplementary fig. S7, Supplementary Material online).

### What Could be the Function(s) of ESEs in Intronless Genes?

Given the evidence that at least some ESEs are likely to be multifunctional, it is relevant to ask what are the splicing-independent functions that they fulfill. A first step toward answering this question would be to identify the protein(s) that bind the motifs that we have identified above as being particularly likely to be multifunctional. However, the task of determining the binding partners of any particular ESE hexamer is not trivial—SR proteins do differ in their binding preferences but there is nevertheless overlap between predicted target motifs (see, for instance, table 1 in Änkö 2014). A further complication is that even though it is usually presumed that ESEs are bound by SR proteins (Blencowe 2000), this assumption is by no means a given for all ESEs in all contexts. Other RNA-binding proteins could therefore also be involved. Nevertheless, a few tentative suggestions can be made.

Several of the motifs that are enriched with smaller mean intron size and/or in intronless genes contain instances of a highly degenerate SRSF2 (the SR protein previously known as SC35) consensus motif (*SSNG*; as determined in Daubner et al. 2012) and also come close to certain other putative target sequences obtained for this protein (Cavaloc et al. 1999; Liu et al. 2000). Strikingly, the *SSNG* motif is present in none of the hexamers that show a significant positive correlation between nucleotide-controlled density and mean intron size. This could, at first sight, make SRSF2 a good candidate for the protein that binds the motifs enriched with smaller intron size and mediates their splicing-independent functions. However, a crosslinking study found SRSF2 binding sites to be enriched in exons flanked by larger, not smaller, introns (Pandit et al. 2013). Some of the more purine-rich motifs also resemble target sites to the SR protein SRSF1 (also known as ASF/SF2), as determined both from in vitro assays (Tacke and Manley 1995; Liu et al. 1998; Ray et al. 2013), and crosslinking and immunoprecipitation studies (Sanford et al. 2009; Pandit et al. 2013).

There is evidence for both SRSF1 and SRSF2 to be involved in various processes in gene expression beyond splicing. For instance, SRSF2 and, less prominently, SRSF1 appear to play a role in transcriptional elongation and have been shown to contact DNA near the transcription start site (Lin et al. 2008; Ji et al. 2013). Mechanistically, these proteins most likely help recruit positive transcription elongation factor b to RNA Pol II, facilitating the release of polymerase paused near the promoter and its entry into the elongation phase (Ji et al. 2013). In addition, both proteins are important for genome stability, as their depletion leads to an accumulation of double strand breaks, most likely through an increased occurrence of R loops (Li and Manley 2005; Xiao et al. 2007; Tuduri et al. 2009).

However, SRSF2 differs from SRSF1 and most other SR proteins because it does not shuttle to the cytoplasm and is instead retained in the nucleus (Cáceres et al. 1998; Cazalla et al. 2002; Sapra et al. 2009). In a previous study, it was observed to show greater binding to an intronless mRNA than several other SR proteins, although it is unclear how generalizable these results are to intronless transcripts in general (Sapra et al. 2009). On the other hand, SRSF1, but not SRSF2, has been shown to enhance translational initiation of specific transcripts by helping recruit the mechanistic target of rapamycin (mTOR) kinase that phosphorylates and thereby represses a translational inhibitor (Sanford et al. 2004; Michlewski et al. 2008; Maslon et al. 2014). It is possible that through this mechanism it regulates the translation of over a thousand mRNAs (Maslon et al. 2014). In addition, SRSF1 is one of the SR proteins for which there is evidence for involvement in the nucleocytoplasmic export of mRNAs (Huang et al. 2003; Lai and Tarn 2004).

An alternative approach to gaining insight into the function of ESEs in intronless genes is to attempt to narrow down the stage of gene expression at which they act. We therefore calculated ESE density in a set of intronless noncoding genes, speculating that if these genes were also enriched in ESE hexamers, then this would suggest that the motifs were unlikely to be preserved primarily for a role in translation-associated processes. No ESE enrichment was found in these genes—instead, they were found to be significantly depleted in ESE hexamers. However, because of serious doubts as to whether the transcripts in question were truly functional or simply the result of spurious transcription, we prefer not to draw any conclusions as to ESE function from this result. An overview of this analysis, along with a tentative hypothesis as to the significance of the depletion signal, is presented in supplementary text S2, figures S10–S16, and table S8, Supplementary Material online.

As a final note, the 4-mer *CCTG* that occurs in several of the ESEs most highly enriched in intronless genes forms part of a consensus motif identified as participating in the nucleocytoplasmic export of at least certain intronless mRNAs (Lei et al. 2013). However, as we found the relevant hexamers to be among the most highly enriched ESEs also in intron-containing mRNAs, which are presumably exported using a different, splicing-dependent mechanism (Masuda et al. 2005), we consider it unlikely that selection to preserve motifs from this particular class of export elements could be driving the enrichment observed for *CCTG*-containing ESEs.

### Why Would Genes that Have a Higher Density of Introns be More Enriched in ESEs?

We also uncovered a positive correlation between nucleotide-controlled ESE density and intron density, a measure defined as the number of introns per base pair of CDS and constructed to simultaneously reflect both exon number and CDS length. It is unclear how to interpret this finding. The decoy splice site model (Wu and Hurst 2015) that posits a greater need for ESEs in exons that are followed by more downstream exon–intron junctions predicts the positive correlation between ESE density and exon number but the negative association with the length of the CDS does not follow as obviously.

Higher intron density implies smaller mean exon size and one explanation could therefore be that only those putative decoys that are within a certain genomic distance of the exon–intron junction that is currently being recognized have the potential to interfere in the process, not the least because the downstream-most introns might not have been transcribed yet. However, given the structure of a typical human gene, the distance to downstream splice sites should depend primarily on the length of the introns and only secondarily on that of the exons, whereas in our data set, normalized ESE density shows a stronger negative correlation with mean exon size than with mean intron size (supplementary spreadsheet S9, Supplementary Material online). We speculate that this is so because longer introns, even though they translate into a greater distance to downstream exon–intron junctions, are more likely to themselves contain cryptic splice sites and that these competing pressures lead to a more complicated overall picture, where some ESEs are more common when introns are small, others when they are large. However, a better understanding of the prevalence and kinetics of cotranscriptional splicing is needed before the data can be interpreted with confidence (Bentley 2014; Mayer et al. 2015; Nojima et al. 2015).

### Controlling for Nucleotide Composition Biases

Throughout the work reported here, a key covariate is the biased nucleotide composition of few-exon genes. As can be seen (fig. 3), there is a gradual trend for genes with *N* introns to have a higher GC_4_ content than those with *N*  + 1 introns, with intronless genes presenting the highest median GC_4_ content. Several previous authors have made similar observations in both animals and plants (Duret et al. 1995; Oliver and Marín 1996; Carels and Bernardi 2000; e.g., Alexandrov et al. 2009; Zhu et al. 2009). More generally, there is a large body of work on the relationship between gene architecture and GC content but the underlying mechanisms remain poorly understood (Duret et al. 1995; Oliver and Marín 1996; Duret and Mouchiroud 1999; Hurst et al. 1999; Carels and Bernardi 2000; Wang and Hickey 2007; Zhu et al. 2009; Tatarinova et al. 2010; e.g., Amit et al. 2012; Glemin et al. 2014).

The issue of how to deal with GC content as a confound in the context of this study is not trivial because its relationship with ESE density is bidirectional. Namely, if a genomic region is AT-rich for reasons unrelated to splice enhancer presence, the probability of observing an ESE simply by chance, independent of any selection pressures acting on the motif, is higher. This is so because of the high frequency of adenine in ESEs (supplementary table S1, Supplementary Material online). On the other hand, selection for a high ESE density will itself modify the base composition. If the latter were the dominant force, the normalization procedures employed in this study would be nonsensical. This is especially true as the set of ESEs used is surely nonexhaustive and the motifs that have been “left out” can be speculated to have a base composition mostly similar to the ESEs already included in the set.

It seems likely, however, that the correlation between GC_4_ and exon number/mean intron size is largely independent of ESE density and that normalizing for nucleotide composition is therefore crucial. The reason for this is that ESE motifs are highly enriched in purines (supplementary table S1, Supplementary Material online). Therefore, if the striking base composition trends in genes with different exon numbers were mainly driven by variation in ESE density, one would expect higher A content to distribute similarly to higher G content but not to higher T content. However, if the base composition skew was primarily one of differences in GC content and was largely independent of ESE density, the opposite pattern should be observed. The latter turns out to be the case: A and T content are both positively correlated with exon number, whereas the correlation is negative for G and C content (supplementary fig. S8, Supplementary Material online). Similar results are obtained when mean intron size is considered instead of exon number (data not shown).

### From Computationally Predicted Motifs to Experimentally Determined Binding Sites

A major caveat of our study, alluded to above, is that it is uncertain how the distribution of computationally predicted ESEs in the genome actually translates into patterns of SR protein binding. The set of ESEs used here probably has a very low false positive rate (because it was defined as the intersection of several previously existing data sets; Cáceres and Hurst 2013) but the false positive rate of the predicted hits is likely to be high. In other words, even though most of the hexamers probably do function as splice enhancers, at least in certain contexts, it is possible that many of the regions where they occur in an mRNA would not actually be bound by SR (or other RNA binding) proteins in vivo, whether it be because of local RNA secondary structure, interference from other proteins binding in the vicinity, or other factors.

Techniques now exist that enable the experimental investigation of genome-wide RNA-protein interactions in vivo through the ultraviolet crosslinking of interacting proteins to RNA, and subsequent reverse transcription and high-throughput sequencing of the RNAs (Licatalosi et al. 2008; Xue et al. 2009; Hafner et al. 2010; Konig et al. 2011). Several such “CLIP-Seq” data sets have been published for SR proteins and all, except for Bradley et al. (2015), that do not discuss the matter, report at least some binding to intronless transcripts (Sanford et al. 2009; Änkö et al. 2012; Pandit et al. 2013; Bradley et al. 2015). The studies largely agree with computational work on ESEs both in terms of wide-scale patterns of site distribution (for instance, the correlation with intron size—Dewey et al. 2006; Cáceres and Hurst 2013; Pandit et al. 2013, although see the results presented in this study) and in terms of the sequence motifs that are uncovered. For example, Sanford et al. (2009), Änkö et al. (2012), and Pandit et al. (2013) all report preferential SR protein binding to purine-rich motifs typical of computationally derived sets of ESEs (although Pandit et al. 2013 also retrieve more pyrimidine-rich motifs for SRSF2).

There is a large discrepancy, however, with regards to the predicted number of sites. According to our data, almost a fifth of the CDS of a typical intron-containing gene overlaps with ESE motifs, a far cry from the sparse and specific binding observed in crosslinking studies. This large difference probably stems, first, from the likely tendency for computational methods that rely on sequence information alone to overestimate the number of target sites and, second, from the low efficiency of CLIP-Seq, a method that probably only captures a fraction of the interactions occurring in the cells at the time of crosslinking (Darnell 2010). The discrepancy might also reflect the possibility that ESE motifs act as a sticky trap for SR proteins. Although any given ESE might not bind an SR protein in a particular transcript at a particular time, in another version of the same transcript it might be the lucky target. Analogy could be made to fly paper, wherein all the fly paper is sticky, but only a small proportion actually has a fly stuck to it.

## Materials and Methods

### General

The UCSC database was queried through a web browser using the UCSC Table Browser (Karolchik et al. 2004; https://genome.ucsc.edu/cgi-bin/hgTables, last accessed 10 June 2015). The Ensembl database (Cunningham et al. 2015; version used for human available from http://dec2014.archive.ensembl.org, last accessed July 3, 2015; version used for mouse available from http://may2015.archive.ensembl.org, last accessed July 3, 2015) was queried using the Python BioServices package, version 1.3.4 (Cokelaer et al. 2013). *R* version 3.0.2. (R Core Team 2013) was used for plotting, for conducting standard statistical tests, or when an *R* package was needed (specified below). All other analysis was performed using custom scripts in Python 3.4.2 (http://www.python.org). Biopython 1.64 (Cock et al. 2009) was used for translating nucleotide sequences into amino acid sequences and for reading/writing phylip files. For other tasks, only the standard library and NumPy 1.9.1. (van der Walt et al. 2011) were used, unless otherwise noted below. GIMP 2.8.14. was used to add letter identifiers and other annotations to supplementary figure S6, Supplementary Material online.

### Retrieval and Filtering of Sequence Data

In order to retrieve intronless genes, the UCSC table browser was queried for all human RefSeq mRNA identifiers with an exon number of 1 (assembly *GRCh38*; group “Genes and Gene Predictions”; track “RefSeq genes,” identifiers matching to NM* only). For intron-containing genes, a similar query was performed but specifying an exon number of 2 or more. The RefSeq mRNA identifiers were then used to retrieve the associated Ensembl gene identifiers from Ensembl (“Ensembl Genes 78,” *Homo* *sapiens* assembly GRCh38), which were then fed back into Ensembl to obtain all the CDSs produced from these genes. This resulted in 2,253 sequences for single-exon genes and 148,633 in the case of multi-exon genes. For intronless CDSs, sequences from genes that did not solely produce single-exon transcripts were excluded (assessed based on the number of chromosomal exon start positions associated with each transcript), while for multi-exon genes, only those particular CDSs that corresponded to single-exon transcripts were removed, while other CDSs from the same gene were retained. The data set was then purged of CDSs that were shorter than 300 bp, contained noncanonical bases, did not start with a start codon, did not end in a stop codon, contained premature stop codons, or were marked as unavailable. If after these filtering steps several CDSs were present from the same gene, only the longest was kept. In the case two CDSs were of equal length, the one that came first alphabetically when comparing their Ensembl transcript identifiers was kept.

The CDSs of the *M.* *mulatta* orthologs of the genes corresponding to the remaining human CDSs were then obtained from “Ensembl Genes 78.” The macaque CDSs were filtered based on reading frame integrity and sequence length similarly to human sequences but without considering exon number. The human and macaque CDSs were then translated into protein and the amino acid sequences aligned using MUSCLE v3.8.31 (Edgar [2004], used via Biopython wrapper). The alignments were then converted back into DNA sequences. The *d_S_* and *d_N_*/*d_S_* values of these alignments were calculated using PAML codeml (Yang 2007) via the Biopython wrapper (Talevich et al. 2012; seqtype = 1, runmode = 0, model = 0, NSsites = []). Those human CDSs that when aligned to the orthologous macaque sequences produced a *d_S_* value above or equal to 0.2 or a *d_N_*/*d_S_* ratio above or equal to 0.5 were removed from the data set to minimize the risk of contamination from pseudogenes (supplementary fig. S9, Supplementary Material online). This final filtering step produced a data set of 344 CDSs for intronless genes and of 10,337 CDSs for intron-containing genes (supplementary table S7, Supplementary Material online).

The CDSs were then BLASTed all against all (for single- and multi-exon genes separately; NCBI local BLAST+ 2.2.30, BLASTN with *e*-value = 10^−^*^4^*; Camacho et al. 2009). The results were used to cluster the genes into paralogous families. A random sequence was picked as seed and all the sequences that had a significant BLAST+ hit to that CDS were added to the same family. All genes that had hits to any of the sequences just added were then also included in the same family. This was repeated until all sequences that had a hit to any of the family members had been included. A sequence outside the family was then randomly picked as the new seed and the process repeated for the next family. This was continued until all genes had been assigned to a cluster. This resulted in 26 nonsingleton families for intronless genes and 1,138 for intron-containing genes. Each family formed one data point, giving rise to a final sample size of 157 single-exon data points and of 5,845 multi-exon data points (supplementary table S7, Supplementary Material online).

### Conducting Readymade Statistical Tests

The R function “wilcox.test()” was used to perform Mann–Whitney *U* tests and R “binom.test()” for binomial tests. R “cor.test()” with “method = ‘spearman’” was used for raw Spearman correlations, whereas the “pcor()” function from the R “ppcor” package (version 1.0; available from https://cran.r-project.org/web/packages/ppcor/), equally with “method = ‘spearman’”, was used for partial correlations. The “rcorr.cens()” function from the R “Hmisc” package with “outx = TRUE” (version 3.16-0; available from https://cran.r-project.org/web/packages/Hmisc/) was used for calculating Goodman and Kruskal’s gamma (the “Dxy” statistic from the “rcorr.cens()” output). In order to estimate the *P* value for gamma, 10,000 simulations were performed where one of the two vectors was randomly shuffled and the statistic calculated using the resulting vector instead of the original, nonshuffled version. This created an empirical distribution from which an effective *P* value could be calculated using $\frac{n+1}{m+1}$, where *n* is the number of simulants presenting a gamma value as high as or higher than that observed with the original vectors and *m* is the total number of simulants. For data points corresponding to nonsingleton families, the family average was used for all statistics in all tests. This resulted in exon number being a floating point number rather than an integer. For plotting only, exon number values were rounded to the nearest integer. Genes with more than 26 exons were excluded from correlation tests that included exon number as a variable so that only exon number classes with at least 50 data points would be considered. This was done to reduce noise as the exon number classes with very few observations predictably showed very high variation in ND (data not shown). Finally, R “p.adjust()” with “method = ‘holm’” was used to perform Holm correction on *P* values. Because several of the variables considered distributed nonnormally, nonparametric tests and statistics are used throughout the analysis.

### Calculating ESE Density

The ESEs used correspond to the intersection set INT3 from Cáceres and Hurst (2013), defined as those motifs that appeared in at least three of the four previously published sets of ESEs. For each CDS, the number of bases that were part of an ESE motif was determined. Bases that were part of two or more overlapping ESEs were only counted once. This number was then divided by the length of the CDS, resulting in the value we term “ESE density.” For nonsingleton families, this statistic was averaged across all family members. The same protocol was followed with the smaller subsets of ESEs. The “high purine,” “low purine,” “high GC,” and “low GC” sets were defined by ordering the motifs in the full INT3 set by GC/purine content and splitting it into two along the median (with motifs that fell on the median assigned to the high GC/high purine set).

### Generating Control Data Set, Calculating Significance and Normalization

In order to control for dinucleotide composition, 10,000 sets of simulated ESEs were generated. The 84 ESE motifs were concatenated and divided into dinucleotides in both reading frames. In total, 10,000 sets of 84 hexamers were then composed by sampling randomly with replacement from this pool of dinucleotides, rejecting those hexamers that belonged to the set of true ESEs. Median ESE density was calculated using each of these simulated sets of ESEs. These medians provided an empirical distribution that was used to compute the effective one-tailed *P* value for the real ESE density observed (calculated as $\frac{n\;+\;1}{m\;+\;1}$, where *n* is the number of simulants with an ESE density as high or higher than the actual ESE density and *m* is the total number of simulants). All other *P* values throughout the entirety of the analysis that were computed from empirical distributions were calculated following the same formula. For analysis requiring normalization of raw ESE densities, the average density obtained for a particular gene over the 10,000 simulations was subtracted from the actual density observed. This difference was then divided by the simulated average, resulting in a measure of enrichment (normalized density or ND). The same protocol was used for smaller subsets of ESEs.

A second simulation control, using shuffled sequences rather than shuffled ESEs, was also tested. The CDSs were divided into subregions of 294 bp, with any leftover bases allocated to the 3′-most subregion, and codons shuffled within each subregion. In this manner, 10,000 simulated versions of both the single-exon and the multi-exon data set were generated and hits to ESEs predicted in these simulants. This created an empirical distribution that could be used similarly to that generated by predicting hits to simulated ESEs. Supplementary table S2, Supplementary Material online, presents initial results from this analysis; however, we chose not to use this method of normalization in the main analysis as it appeared to be less efficient at normalizing out GC content biases (data not shown). We were also concerned that even if the shuffling was performed within smaller subregions of full CDSs, aspects of the highly nonrandom base composition along the CDS (Tuller and Zur 2015) would still not be captured in the simulants, rendering dubious the biological well-foundedness of the simulations.

### Calculating D_s_ within ESE Regions

For each data point (a random member was picked from nonsingleton families), the alignment to macaque that produced the lowest *d_S_* during initial filtering was selected. All non-ESE regions were then removed from the alignment. If needed, each block of contiguous ESE bases was modified in such a way as to be entirely composed of full codons so that *d_S_* could be calculated. To achieve this, the final codon of the block was trimmed if it was incomplete, while the region was expanded to include 1 or 2 non-ESE bases upstream if the first codon was incomplete. If an incomplete initial codon coded for Leucine or Arginine, the first two bases were replaced by *GA* if the last base was 2-fold degenerate and *GC* if it was 4-fold degenerate. The same changes were made to the macaque sequence, unless if the corresponding codon in macaque was an indel, in which case no changes were made in macaque. This enabled the maintenance of the final synonymous site of the codon (that was ESE) without introducing a non-ESE synonymous site at the first base.

These alignments were then concatenated across all CDSs (for intronless and intron-containing genes separately) and the *d_S_* calculated using PAML “codeml” as had been done previously for full CDSs. The same protocol was repeated using each of the 10,000 simulated sets of ESEs (for computational reasons, only 1,000 simulants were used for intron-containing genes). The *d_S_* values thus obtained formed an empirical distribution used for computing an effective *P* value for any decrease in *d_S_*. A normalized *d_S_* value was also calculated by subtracting the simulated average from the actual *d_S_* rate and dividing the difference by the simulated average.

### Calculating SNP Density

The positions of all “dbSNP” synonymous SNPs annotated as mapping to any of the CDS examined were obtained from Ensembl (“Ensembl Variation 78”). For either data set (single- or multi-exon genes), the total number of SNPs at 4-fold degenerate sites within ESE regions was divided by the total number of 4-fold degenerate sites in ESE regions. This analysis was also performed using ESE regions derived from the simulated data sets and the resulting distribution used for calculating an effective *P* value. A normalized SNP density value was calculated as above for normalized *d_S_*.

### Calculating the Fraction of SNPs with a Low MAF

The positions and MAFs of all 1000Genomes SNPs (synonymous and nonsynonymous) annotated as mapping to any of the CDSs examined were obtained from Ensembl (Ensembl Variation 78). The number of SNPs in ESEs that had an MAF below 1/2,000 (threshold determined based on what gave the most significant result for multi-exon genes, which we considered as a positive control) was divided by the total number of SNPs in ESEs. This was also done for simulated ESE motifs, thus creating an empirical distribution.

### Determining the Density of Particular ESEs in Mean Intron Size Bins

ESE motifs were predicted in CDSs as described above, except that instead of calculating overall ESE density, a vector was returned for each CDS that contained the number of bases that overlapped instances of each particular ESE in that sequence. Genes were grouped into families as described above and mean intron size and the overlapping base counts for each ESE motif averaged within families, mean intron size having been calculated based on exon chromosome start positions that had been retrieved from Ensembl BioMart. The data points were then binned by mean intron size. The binning was necessary because many genes contain no occurrences of a particular ESE motif and the resulting abundance of zero density data points could have rendered analysis difficult and noisy. Intronless genes, which have an intron density of 0, formed the first bin, while intron-containing genes were divided along every 1/48^th^ quantile. This resulted in 49 bins, indexed from 0, with near-equal sample sizes in the intronless bin and each of the intron-containing ones (a total of 38 bins were used when putative recent retrocopies were included in the analysis so as to maintain the uniformity in sample sizes). Next, the overlapping base counts were summed across mean intron size bins, for each ESE separately, and divided by the sum of the lengths of the CDSs in each particular bin. This gave a single measure of density for each ESE and each mean intron size bin. These values were stored in a 49 × 84 matrix with mean intron size bins in the rows and ESEs in the columns.

To control for nucleotide composition biases, 60 simulated versions of each ESE were generated. To do so, we made a list of the five dinucleotides that appeared in each ESE (in the two reading frames) and generated all the possible hexamers that could be constructed from these dinucleotides, which amounted to 60 control motifs for each ESE. This allowed for 60 simulated versions of the ESE data set. The above analysis was then performed using each simulated set. We thereby obtained 60 additional 49 × 84 matrices with intron density bins in the rows and ESEs in the columns, as above. For each position of the matrix, we then took the median of the values that appeared at that position throughout the simulations. We then subtracted that median from the value appearing at that position in the true matrix and divided the difference by the simulated median, resulting in a version of the matrix that was normalized for dinucleotide composition biases.

Importantly, the simulated hexamers generated for each ESE could include motifs that already belonged to the INT3 set. This means that those ESEs that had a dinucleotide composition that more closely resembled the overall dinucleotide frequencies observed in ESEs (notably purine-rich motifs) were penalized more severely by the normalization procedure. This issue is unlikely to be problematic for the correlations between density and mean intron size bin indices, as the biases would have been similar across all sequences, meaning that any skews in the distribution of ESEs across mean intron size bins should be unaffected. It could, however, affect the analysis in figure 7 where we order ESEs based on their ND in either intronless or intron-containing genes. In order to estimate the probability that this issue could be seriously biasing our work, we calculated for each ESE the fraction of its 60 simulants that corresponded to actual ESEs in the INT3 set. We then performed a partial Spearman correlation between this measure, the correlation coefficient between the ND of a motif and mean intron size bin indices, and the ND of that motif in either intronless or intron-containing genes (taking the median of all intron-containing mean intron size bins in the latter case). We found that the correlation between the ND of the motif and the correlation coefficient with intron size bin indices remained significant even after controlling for the fraction of simulants that were part of INT3 (intronless genes: ρ ≈ −0.753, *P* ≈ 7.634 × 10^−^^25^; intron-containing genes: ρ ≈ −0.370, *P* ≈ 3.379 × 10^−^^4^). The bias therefore seems to have some effect on the results but is unlikely to explain the totality of the effect.

### Comparing ESE Density in Exon Flanks and Exon Cores

The Ensembl transcript identifiers of all the sequences in our intron-containing data set were fed to Ensembl to retrieve the associated exons and their associated chromosomal start positions. For nonsingleton paralogous families, a random transcript identifier was picked from each. Exons from the gene *ENSG00000183091* (“*nebulin*”) were excluded as the gene has many identical exons that could create a problem of statistical nonindependence. Terminal exons and exons that were not entirely part of the CDS were also removed. As a final filter, those exons that were shorter than 211 bp were also removed. This was done because we wished to extract three 69-bp subdivisions from the exon (upstream flank, core, and downstream flank) and would potentially have to exclude up to two bases from either end of the exon to make sure that both ends were in frame (69 × 3  +  2 × 2 = 211). The exons were then mapped to the corresponding open reading frame (ORF) in order to determine their reading frame. The first 69 bp of the exon (counting from the 5′ end), starting with the first full codon, was then defined as the upstream flank, while the last 69 bp, ending with the last full codon, was defined as the downstream flank. If after subtracting 69, the number of bases (and thus the number of codons) separating the two flanks was divisible by 2, the exon core was defined as the 69 bp exactly midway between the upstream and the downstream flank. Otherwise, it was defined as the 69 bp that was at a distance of *n* nucleotides from the end of the upstream flank and of *n* *+* 3 nucleotides from the start of the downstream flank. ESE density was then computed as for full ORFs when hits were predicted to all ESEs. When hits were only predicted to a subset of ESEs, then instead of calculating the median density per gene, the total number of bases overlapping an ESE was summed across sequences. That sum was then divided by the total length of the sequences and the same procedure repeated with the 10,000 sets of simulated hexamers. Using this protocol was necessary because the sequences are short and therefore, when hits are only predicted to a few ESEs, most sequences will present no hits, giving rise to a median density of 0 for both true ESEs and for simulants.

### Retrieval and Analysis of Mouse (*Mus musculus*) Sequences

The mouse data set was prepared similarly to the human data set, except that rather than being retrieved directly from Ensembl, the CDSs were extracted from the genome sequence (*GRCm38*) based on Ensembl annotations (release 80) using bedtools (Quinlan and Hall 2010). Only sequences assigned to particular locations on particular chromosomes were retained (that is to say, sequences that mapped to unmapped contigs were discarded). Unlike when preparing the human data set, no filtering was performed based on the length of the CDS. The CDSs that passed the tests for ORF integrity were aligned to rat orthologs as determined by Ensembl (*Rattus norvegicus*, assembly *rn6*). Sequences for the latter were extracted based on Ensembl annotations (release 80) similarly to what had been done for mouse, except that CDSs on unmapped contigs were also retained. Those mouse sequences that had at least one ortholog to which they aligned with a *d_N_*/*d_S_* ratio below 0.5 and a *d_S_* rate below 0.3 were retained. The sequences were then clustered into paralogous families and ESE density (both of the full INT3 set and of individual motifs) calculated as for human. The *d_S_* analysis was also identical to that performed in human, with the rat ortholog that aligned to the mouse CDS with the lowest *d_S_* being used to determine the rate of evolution at synonymous sites.

### Identifying Retrocopies

The UCSC Table Browser was used to obtain the coordinates of putative retrocopies in the human genome (assembly “Dec 2013 (GRCh38),” group “Genes and Gene Predictions,” track “Retrogenes v9,” table “ucscRetroAli9”). The Ensembl database was then used to determine the chromosomal coordinates of the genes in the intronless set. Those 82 intronless genes that overlapped by at least 50% with a putative retrocopy region were defined as the “broad retrocopies set.” Next, the parent genes of these retrocopies, as indicated by the UCSC Table Browser, were examined. Those parents that had identifiers that did not start by *NM* (were not verified RefSeq mRNAs) were removed. The remaining RefSeq parent identifiers were then given to Ensembl as input to obtain the corresponding Ensembl gene identifiers and CDSs. A single RefSeq identifier typically corresponds to several Ensembl CDSs, all of which were retrieved. These parent CDSs were then filtered to remove sequences with nonstandard bases, which were not a multiple of three long or contained premature stops (without this filtering step, the sequences could not have been translated into protein).

The remaining sequences were then matched to their putative retrocopies within the intronless set. Only those pairs were kept where the length of the shorter CDS was over 50% of the length of the longer CDS. Finally, both the retrocopies and their putative parents were translated into protein sequences, aligned using MUSCLE, converted back into DNA and the *d_N_* of the resulting DNA alignment calculated using PAML. Those putative retrocopies that aligned to at least one of their associated parent CDSs with a *d_N_* below 0.2 were kept and their lowest *d_N_* match designated as their parent (the threshold was defined empirically based on what seemed to best distinguish between false and true positives). These remaining 21 intronless genes formed the “strict retrocopies set.” Finally, in order to perform the analysis reported in supplementary text S1, Supplementary Material online, the strict retrocopies set was examined for the presence of several genes from one paralogous family, as defined during the initial preparation of the intronless data set. In such cases, the gene that aligned to its parent with the lowest *d_N_* was kept. This resulted in a final set of 18 data points for the analysis in supplementary text S1 and spreadsheet S1, Supplementary Material online.

### Comparison of Retrocopies and their Parents (Results Reported in supplementary text S1, Supplementary Material online)

ESEs were predicted in both the 18 retrocopies (strict set after removal of paralogs) and their putative parents using both the actual ESE motifs and the 10,000 simulated sets, and storing for each hit the position at which the first base of the ESE hexamer matched in the CDS. The raw ESE densities and ND (calculated as above) were then compared between the two sets by a paired Wilcoxon signed-rank test. The same test was also performed to compare the GC content of the parent and retrocopy sequences.

The Ensembl API was then used to retrieve the exon sequences of the parent transcripts. For each noninitial exon of a parent transcript, the first 30 bp were mapped to the parent CDS (if possible), so as to determine the positions of exon–exon junctions. These were then mapped to the paralogous region in the corresponding retrocopy, allowing us to define which positions in the modern intronless gene were likely to correspond to old exon–exon junction positions. If less than 50% of the region in the retrocopy that aligned to the first 30 bp of the parent exon was intact (that is to say, was not constituted of indels), that exon–exon junction was only retained among the parent exon–exon junctions but not among the retrocopy pseudojunctions. We then calculated, for parents and for retrocopies separately, how many of the ESE hexamers that had a hit in a given sequence were located within 50 bp of (what used to be) an exon–exon junction, as judged by the position of the first base of the ESE. This count was then divided by the total number of ESE hits in the sequence, resulting in a ratio for each CDS. These ratios were compared between parents and retrocopies using a paired Wilcoxon signed-rank test.

### Calculating ESE Density, d_S_, and SNP Density without Retrocopies and in Broad Set Retrocopies Only

Two new data sets of intronless genes were prepared, one in which broad set retrocopies had been removed and one consisting of broad set retrocopies only. The sequences were then aligned to macaque and clustered into families as described above for the full data set. All analyses were then carried out on these two data sets as they had been previously on the full set of intronless genes.

### Simulations to Estimate the Importance of Putative Retrocopies

ND in the full set of intronless genes was calculated as previously, except that only 1,000 simulated sets of ESEs were used as control instead of the usual 10,000 (this was done so that the results would be comparable with those obtained in the simulations described below, where using 10,000 controls was not feasible for computational reasons; see supplementary table S3, Supplementary Material online, for results). The broad set retrocopies were then removed and the analysis repeated on the resulting reduced data set (using the same clustering into families that had been obtained for the full data set). The resulting reduction in ND and the increase in *P* value were recorded. As the next step, 1,000 simulations were performed where 82 (mimicking the broad retrocopies set) random genes were removed from the intronless data set and the change in ND and *P* value, when compared with the full data set, recorded. This created an empirical distribution that could be used to calculate a one-tailed *P* value to estimate the significance of any changes in ND or *P* value observed when putative retrocopies were removed.

The protocol used for simulating changes in SNP density after removal of retrocopies was identical to that used with ESE density, except that SNP density was calculated instead of ESE density (supplementary table S6, Supplementary Material online).

### Analysis on Long Noncoding (Lnc)RNAs (Results Reported in supplementary text S2, Supplementary Material online)

Dataset S6, composed of those intergenic lncRNAs that had been found to be expressed at significantly higher levels than expected from random simulations, was retrieved from the supplementary information of Hangauer et al. (2013). The data set was filtered to remove records that did not have strand information and the remaining coordinates converted to *hg38* using the UCSC Genome Browser liftOver tool (Kent et al. 2002). Putative intronless lncRNA transcripts were then isolated, the corresponding sequences extracted from the genome using bedtools, and then clustered into families as had been done for protein-coding genes.

In order to assess the conservation of the sequences, the phastCons scores (Siepel et al. 2005) of the regions corresponding to the presumed intronless lncRNAs, as well as those corresponding to the set of CDSs from intronless protein-coding genes (excluding broad set retrocopies) were retrieved from the UCSC Genome Browser (group “Comparative Genomics,” Track “Conservation”, Table “phastCons100way”). Sequences where less than 75% of the bases had associated phastCons scores were discarded. For the sequences that remained (the vast majority), the mean phastCons score was calculated over all the positions for which the score was available. For each putative lncRNA, phastCons scores were similarly extracted and averaged for an equally sized region immediately 3′ of the lncRNA. Two “conserved” subsets of intronless lncRNAs were then set aside—one constructed by filtering out all those lncRNAs whose mean phastCons score was lower than the lowest mean score observed in intronless CDSs (≈0.095), the other by removing those lncRNAs that had a lower mean score than an equally sized region immediately downstream of the lncRNA. The two conserved sets were then equally clustered into paralogous families.

In order to better assess whether or not it was likely that the purported lncRNAs were mere results of spurious transcription, 1,000 random regions of 1 kb were selected from the genome (using the bedtools “random” command). Regions that included *N* bases were discarded, leaving a total of 949 sequences. The phastCons scores for these regions were retrieved and averaged and their ESE density calculated as above, with the sole difference that the sequences were not clustered into paralogous families.

Several additional manipulations were then performed to help interpret the ESE depletion in random regions that had been revealed by the steps described in the previous paragraph. First, over 100 iterations, 100 new random 1-kb-long regions were selected from the genome and those that included *N* bases removed. ESE density was calculated each time, creating an empirical distribution of raw and normalized ESE density values. This was also done separately in the intergenic, intronic, exonic, and coding regions of the genome, as defined by Ensembl annotations (release 82). In these latter simulations, the sequences were clustered into families, whereas they were not for any of the analyses performed in the rest of this section. Both in this simulation and in all others to be described in this section, 1,000 simulations were used instead of the usual 10,000 to determine the *P* value associated with each estimate of motif density obtained. Second, over 100 iterations, 84 sets of completely random hexamers were generated and their density calculated in a set of 100 random genomic regions. Third, the overall frequencies of the four bases in the human genome were calculated. At each of the 100 iterations, a set of 84 hexamers was then generated, with the probability of any particular base being used at a given position corresponding to its frequency in the genome. These hexamers were then used to scan the same set of 100 random 1-kb-long regions each time. Finally, a third simulation similar to the previous two was performed, except that this time the motifs used corresponded to a different set of 84 random 6-bp-long regions picked from within CDSs. These simulations were performed using a mixture of custom scripts and the bedtools “shuffle” command.

Over 100 iterations, the density of INT3 ESEs was also determined in random 1-kb-long regions randomly picked from the repeatmasked genome (available from http://www.repeatmasker.org/, last accessed December 6, 2015). Sequences containing *N*s were discarded. Because this would have resulted in a smaller actual number of sequences than in the case of regions picked from the non-repeatmasked genome (because of the greater frequency of *N* bases in the repeatmasked sequence), the initial number of random regions picked was 2,800 rather than 100. This resulted in a little over 100 remaining sequences on most iterations. Finally, the coordinates of long interspersed nuclear element (LINE), short interspersed nuclear element (SINE), and long terminal repeat (LTR) retrotransposons were obtained using the UCSC Genome Browser (group “Repeats,” track RepeatMasker, table “rmsk”). For each, 100 iterations were performed where a different set of 1,000 300-bp-long regions were picked from within these coordinates and INT3 ESE density calculated within each set.

Note that the plots resulting from this part of the analysis (supplementary figs. S10–S16, Supplementary Material online) were created using the Python matplotlib library version 1.4.2. (Hunter 2007) rather than R.

## Supplementary Material

Supplementary table S1–S8, figures S1–S16, texts S1 and S2, and spreadsheets S1–S9 are available at *Molecular Biology and Evolution* online (http://www.mbe.oxfordjournals.org/).

Supplementary Data
