# Supplementary material for: Purifying Selection on Exonic Splice Enhancers in Intronless Genes
Source: Mol Biol Evol. 2016 Jan 23;33(6):1396–418. doi: 10.1093/molbev/msw018 (PMC4868121; doi:10.1093/molbev/msw018)
Supplement: Supplementary Data [file supp_33_6_1396__index.html]

Purifying Selection on Exonic Splice Enhancers in Intronless Genes — Purifying Selection on Exonic Splice Enhancers in Intronless Genes — Supplementary Data 

# Purifying Selection on Exonic Splice Enhancers in Intronless Genes

## Supplementary Data

files

- Supplementary Data - xls file
- Supplementary Data - pdf file
