# Supplementary material for: Purifying Selection on Exonic Splice Enhancers in Intronless Genes
Source: Mol Biol Evol. 2016 Jan 23;33(6):1396–418. doi: 10.1093/molbev/msw018 (PMC4868121; doi:10.1093/molbev/msw018)
Supplement: Supplementary Data [file supp_msw018_ESE_avoidance_paper_Supplement_merged.pdf]

Supplementary material

Supplementary Spreadsheets

Supplementary Spreadsheet 1: genes considered as broad set retrocopies, strict set retrocopies and the strict retrocopies set after removal of paralogs.

Supplementary Spreadsheet 2: ESEs considered as high GC, low GC, high purine or low purine.

Supplementary Spreadsheet 3: Results from a Spearman rank correlation between normalized/raw ESE density and mean intron size for different subsets of ESEs.

Supplementary Spreadsheet 4: Raw density of different ESE motifs in genes binned according to their mean intron size (*hg38*).

Supplementary Spreadsheet 5: Normalized density of different ESE motifs in genes binned according to their mean intron size (*hg38*).

Supplementary Spreadsheet 4: Raw density of different ESE motifs in genes binned according to their mean intron size (*mm10*).

Supplementary Spreadsheet 5: Normalized density of different ESE motifs in genes binned according to their mean intron size (*mm10*).

Supplementary Spreadsheet 8: ESE motifs the normalized density of which is significantly correlated with mean intron size bin indices, along with the associated correlation coefficients and *p*-values.

Supplementary Spreadsheet 9: Results from a partial Spearman correlation between normalized ESE density, mean exon size and mean intron size.

Supplementary Text 1

After the several steps of filtering described in the *Materials and Methods*, we obtained a set of 18 putative intronless retrocopies and the CDS of their likely intron-containing parents. We then proceeded to characterize these sequences with hopes of gaining insight into the fate of ESEs in a gene that suddenly finds itself deprived of its introns. Because of the very small sample size ($n = 18$), however, we prefer to provide only a description of the dataset and to refrain from making inferences.

Median ESE density was found to be only marginally lower in the retrocopies than in their parents (≈0.165 vs ≈0.167; Supplementary Figure 4), although the difference is greater after normalization (≈0.260 in the retrocopies *versus* ≈0.285 in parents). The latter is true despite the higher median GC_4 of the retrocopies (≈0.576 *versus* ≈0.496). We also calculated the percentage of ESEs in the retrocopies that were within 50 bp of where we presume the old exon-exon junction to have been (see *Materials and Methods* for details) and the same for their parents and their actual exon-exon junctions. The median value obtained was, expectedly, higher in the parents (≈0.670) than in the retrocopies (≈0.595).

Supplementary Text 2

We hypothesized that if the ESEs in intronless protein-coding genes were mainly involved in nuclear processes, they should also be enriched in intronless long non-coding (lnc)RNAs. Conversely, if their roles were mainly translation-related, no enrichment should be observed in intronless genes that do not encode for proteins. ESEs are known to be under purifying selection in multi-exonic lncRNAs (Schüler et al., 2014) but to our knowledge, there has been no work on ESE motifs in intronless lncRNAs.

To test this hypothesis, we retrieved the set of intergenic lncRNAs published in Hangauer et al. (2013) (dataset S6) and kept only those records for which information was available as to the strand of transcription. We then divided the set of putative lncRNAs into two based on whether or not the proposed genes contained introns and for either subset, clustered the transcripts into paralogous families as had been done for protein-coding genes. In addition, because of concerns that many of the proposed intronless lncRNAs could merely be products of spurious transcription, we also applied two different conservation filters to the full set of intronless lncRNAs, thus creating two smaller subsets that were more conserved and presumably more likely to be functional. The first filter isolated those lncRNAs whose mean phastCons score (obtained from the UCSC Genome Browser *Conservation* track and averaged over the length of the gene) was higher than or equal to the lowest mean phastCons score observed in our set of intronless CDS (without broad set retrocopies) (Siepel et al., 2005). We will refer to this subset as *conserved (CDS).* The second subset − *conserved (3')* − was made up of those intronless lncRNAs that presented a mean phastCons score that was greater than that of an equally sized region directly 3' of the purported transcript.

The median ESE density in each set was then calculated and the significance of the estimate determined as described for CDSs. Surprisingly, not only was there no enrichment of ESEs in any of the datasets, the full set of intronless lncRNAs was actually found to be significantly *depleted* in ESEs, with the two conserved sets also exhibiting depletion, although without quite reaching significance (see Supplementary Table 8).

At first sight, these results could be taken to imply that the ESEs in intronless genes are acting in a translation-related role, however, this conclusion is most likely premature. Distinguishing between functional lncRNAs and those that are merely spurious results of pervasive transcription is not a trivial task and might be even more difficult in the case of intronless transcripts. In order to assess the probability that the intronless lncRNAs were functional, we randomly picked 949 regions of 1kb from anywhere in the human genome, with the sole condition that they could not contain *N* bases, and determined ESE density and phastCons scores also for this set. We found that the dataset of presumed intronless lncRNAs was indistinguishable from the random set both in terms of ESE density (Supplementary Table 8) and in terms of conservation (or, to be more exact, the random set actually had *higher* mean phastCons scores, suggesting that the presumed lncRNAs were evolving *faster*; median of mean scores for putative intronless lncRNAs: ≈0.039, median of mean scores for random regions: ≈0.056, *p*-value from Mann-Whitney *U*-test: $\approx 3.643 * 10^{-5}$). We therefore conclude that we cannot draw inferences as to the function of ESEs in intronless protein-coding genes from this analysis because we cannot have confidence that at least a significant proportion of our intronless lncRNAs are functional. Because of the similarity between the results obtained with the full set and the two conserved subsets in terms of ESE density, we prefer not to draw conclusions from the conserved subsets either.

There remains nevertheless the problem of how to interpret the fact that ESEs appear to be depleted in random genomic regions. Crucially, we observe not a simple lack of enrichment but rather that the motifs are significantly less common than would be expected given the underlying nucleotide composition. A full investigation of the issue is outside the scope of this paper but we have nevertheless taken a few preliminary steps to better understand the problem.

We first hypothesized that the depletion could reflect a methodological artifact of some sort inherent in the density calculations. This, however, seems unlikely as the effect is not observed for completely random hexamers (Supplementary Figure 10) or for random hexamers constructed to match the genome mononucleotide composition (Supplementary Figure 11), both of which seem to distribute according to random expectations. Moreover, the depletion in ESEs is observed more or less consistently

across many different sets of random regions (Supplementary Figure 12). It therefore appears that the depletion is genuine.

Our second hypothesis was that perhaps, the depletion was not particular to ESEs and was rather a reflection of more general differences in, say, trinucleotide composition between coding regions and the rest of the genome. In this case, not only ESEs but most hexamers commonly found in protein-coding regions should be depleted in random regions. This prediction turned out to be incorrect: sets of random hexamers picked from inside coding regions tend to be either enriched (when compared to a nucleotide-controlled control) in random regions or to occur at levels corresponding to random expectations (Supplementary Figure 13). The depletion is therefore likely specific to ESE hexamers.

We next sought to determine whether the depletion could be particular to specific subregions of the genome, for instance those deriving from transposable elements. We found that although random regions sampled from retrotransposons (Supplementary Figure 14) were indeed depleted in ESEs, random non-*N*-containing regions from the RepeatMasked genome (Smit et al., 2013-2015), that should be mostly devoid of retroelements, also exhibited a lower than expected density (Supplementary Figure 15), suggesting that the effect was not specific to transposable elements. Finally, we asked whether the depletion could be specific to intronic regions, as one could imagine how for certain splice factors, extensive binding to introns could interfere with splice regulation. This prediction also proved inaccurate: both intergenic and intronic regions are significantly depleted in ESEs, with intergenic regions showing a significantly stronger effect ($p \approx 5.936 * 10^{-16}$ from Mann-Whitney *U*-test comparing median ND values between 100 sets of random regions sampled from either the intergenic or the intronic parts of the genome; Supplementary Figure 16). These results would imply that the ESE depletion is a general property of non-exonic regions rather than a phenomenon that is specific to particular subregions.

In conclusion, it therefore seems that the under-representation of ESEs that we have observed in random genomic regions is a genuine biological phenomenon, that it is particular to ESEs and that it is a global property of the non-exonic part of the genome (or at least that we have not been able to find evidence of certain regions

being more concerned than others). Explaining this surprising finding is a difficult task. That the binding of certain RNA-binding proteins to spurious transcripts could be deleterious is not inconceivable. For instance, varying the quantities of various RNA-binding proteins in particular cell types at particular times seems to be a mechanism for alternative splicing regulation (Grosso et al., 2008; Han et al., 2011; Hanamura et al., 1998). In this context, it is easy to imagine how products of spurious transcription, were they to sequester away significant quantities of such a protein, could perturb splice regulation. However, it is harder to conceive that a single ESE in a weakly transcribed intergenic region of the genome could have a sufficient effect on the availability of its binding partner that the deleterious consequences would be so pronounced as to be visible to selection, especially given the low effective population size in humans (Lynch and Walsh, 2007). It therefore seems unlikely that the pattern of depletion observed could have been created through selection acting on point mutations across the genome.

We therefore propose an alternative explanation: the depletion of ESEs in random genomic regions could derive not from selection acting on intergenic/intronic regions but rather on the RNA-binding proteins themselves. Namely, proteins that would chiefly be expected to bind in exons could have been selected to recognize motifs which happened to be particularly common in exonic regions (perhaps because they represented hexamers that coded for common pairs of amino acids) and particularly uncommon elsewhere so as to minimize the extent of unnecessary binding to products of spurious transcription. This would also have an added benefit: if the binding of a protein is to help define a particular region as being exonic, tuning the binding preferences of the protein to pre-existing sequence biases of exonic regions would help prevent potentially deleterious binding to introns.

It should be stressed that the likelihood of this scenario has not been tested in any way and that it therefore remains highly hypothetical. It should also be noted that this hypothesis, if true, could explain the ESE enrichment in intronless genes − if the hexamers that, say, SR proteins bind to were enriched in exons even before they became target motifs for the protein then there is no reason to expect that they would not be enriched also in single-exon genes. However, it could not explain the purifying

selection that we observe in ESEs in intronless genes. An explanation evoking functional significance therefore still remains necessary.

Supplementary Figures

Supplementary Figure 1

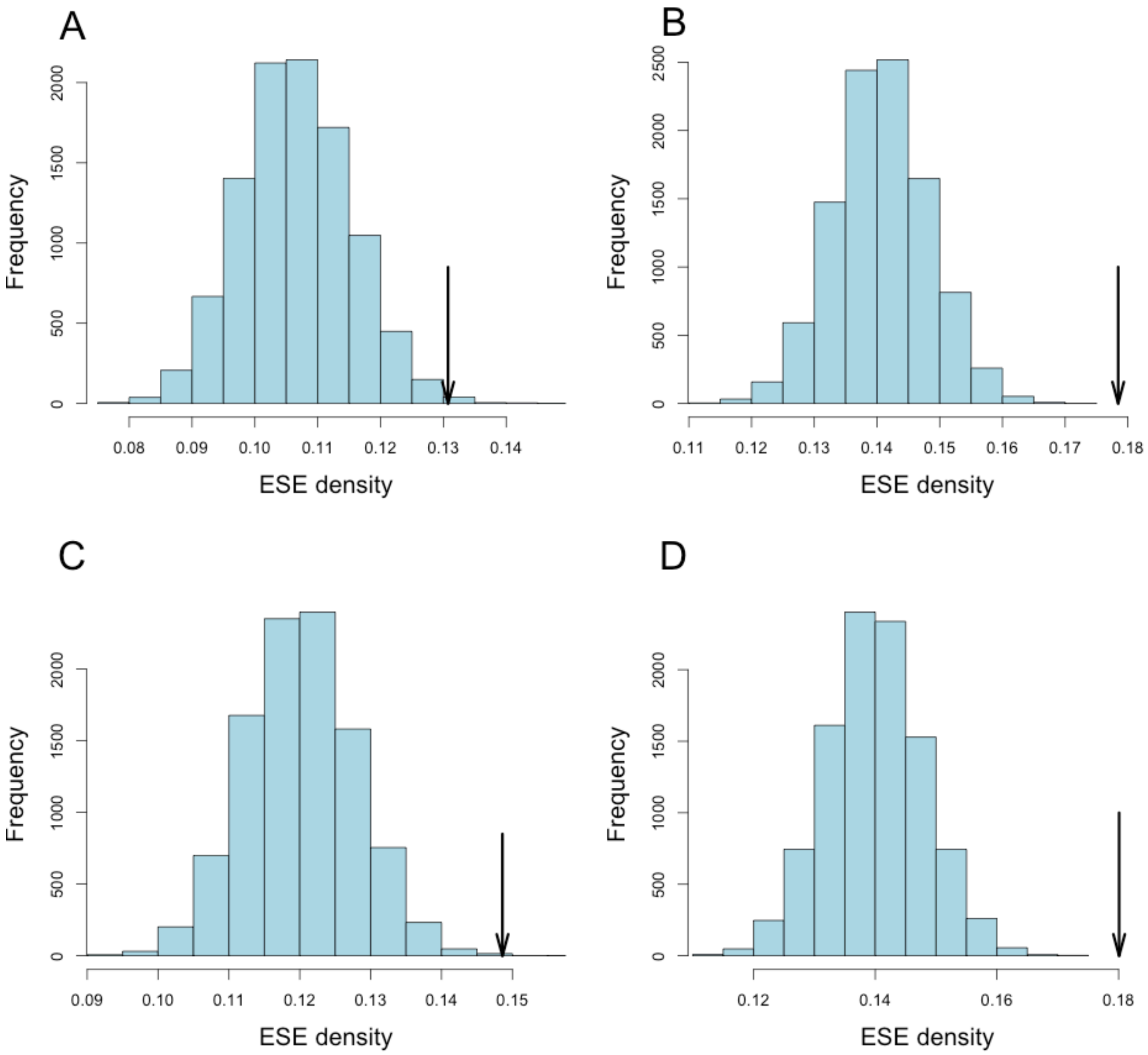

Supplementary Figure 2

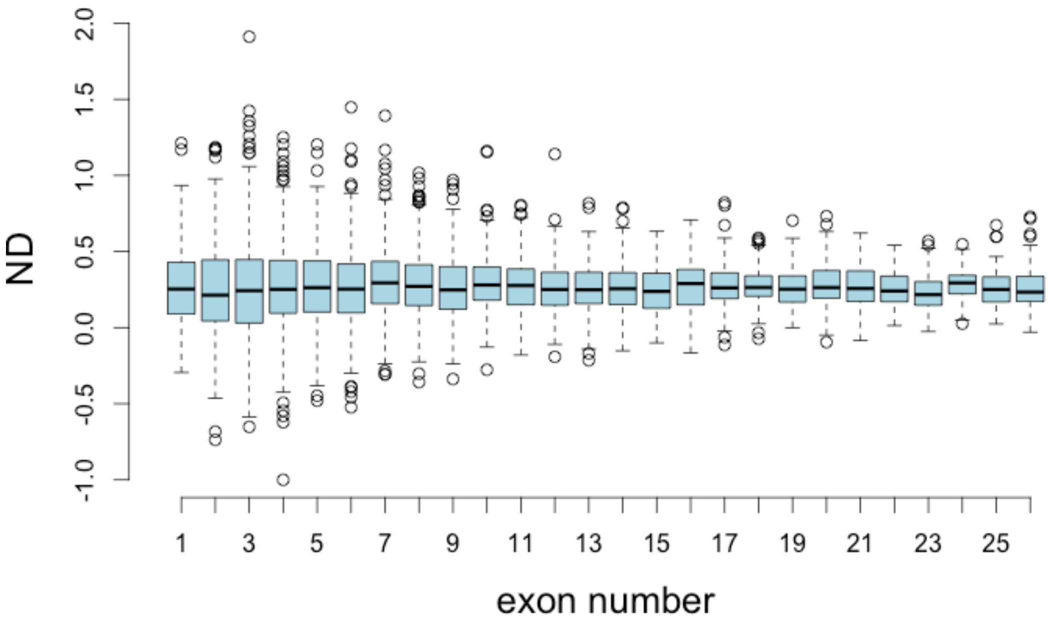

Supplementary Figure 3

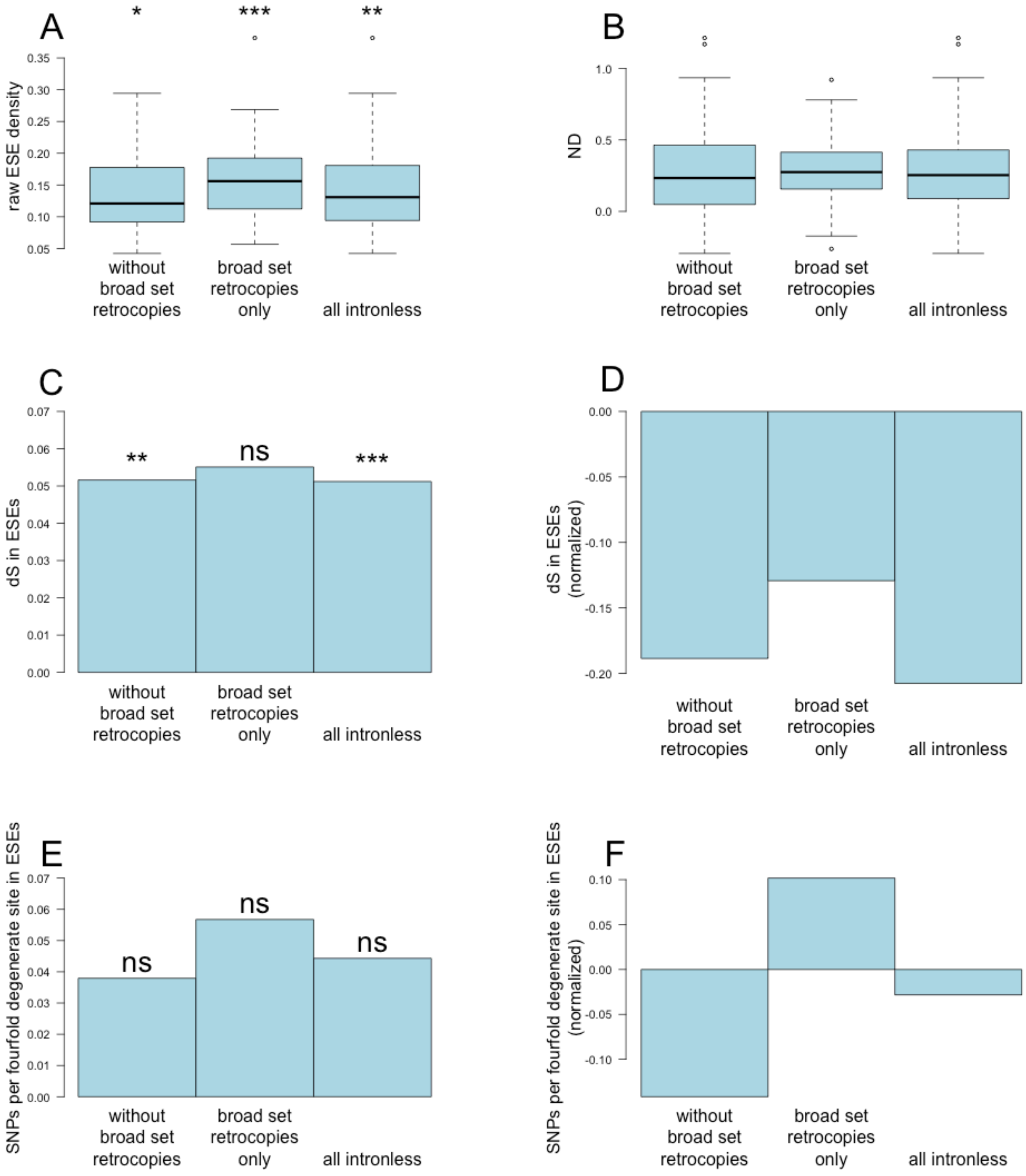

Supplementary Figure 4

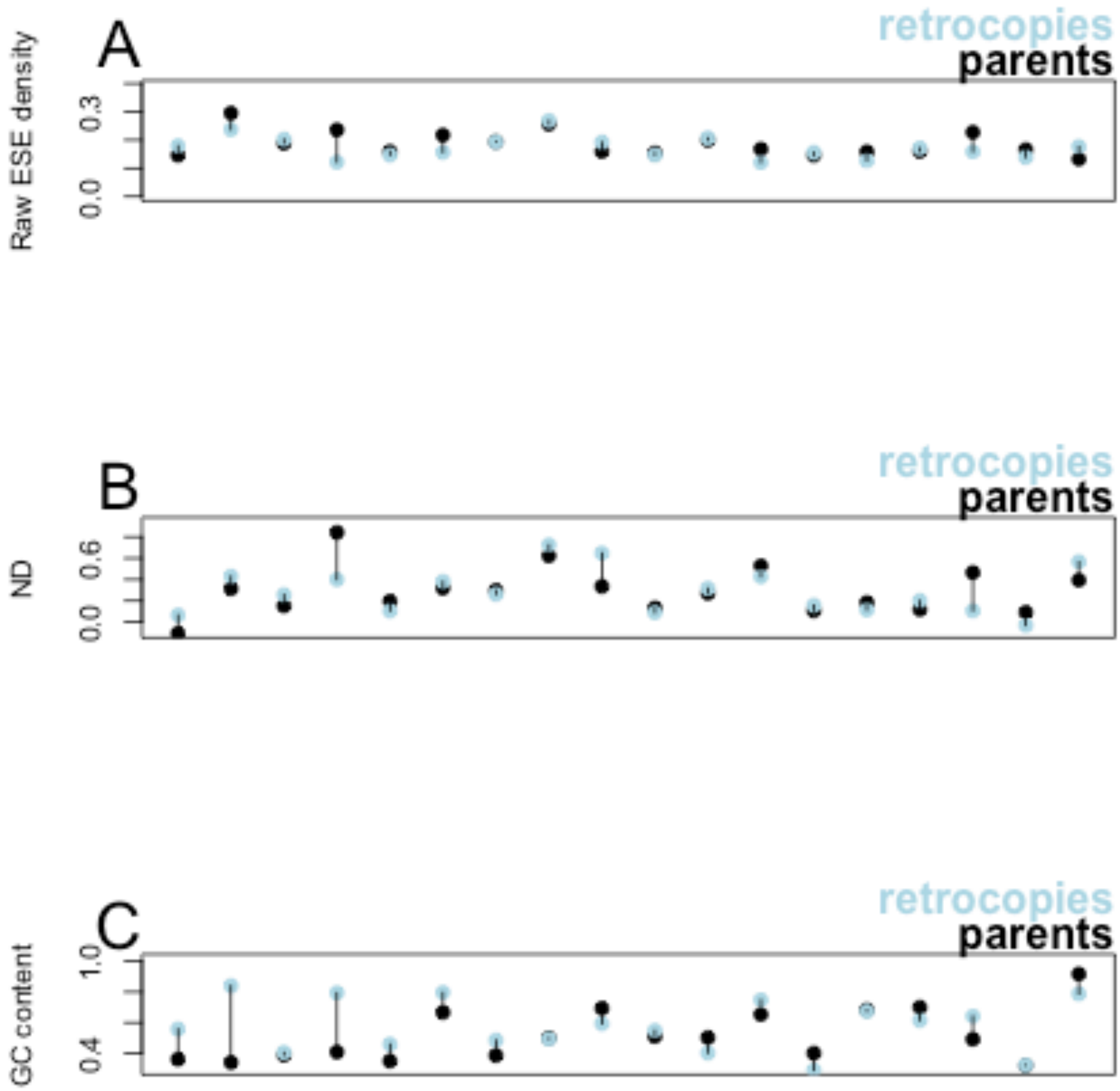

Supplementary Figure 5

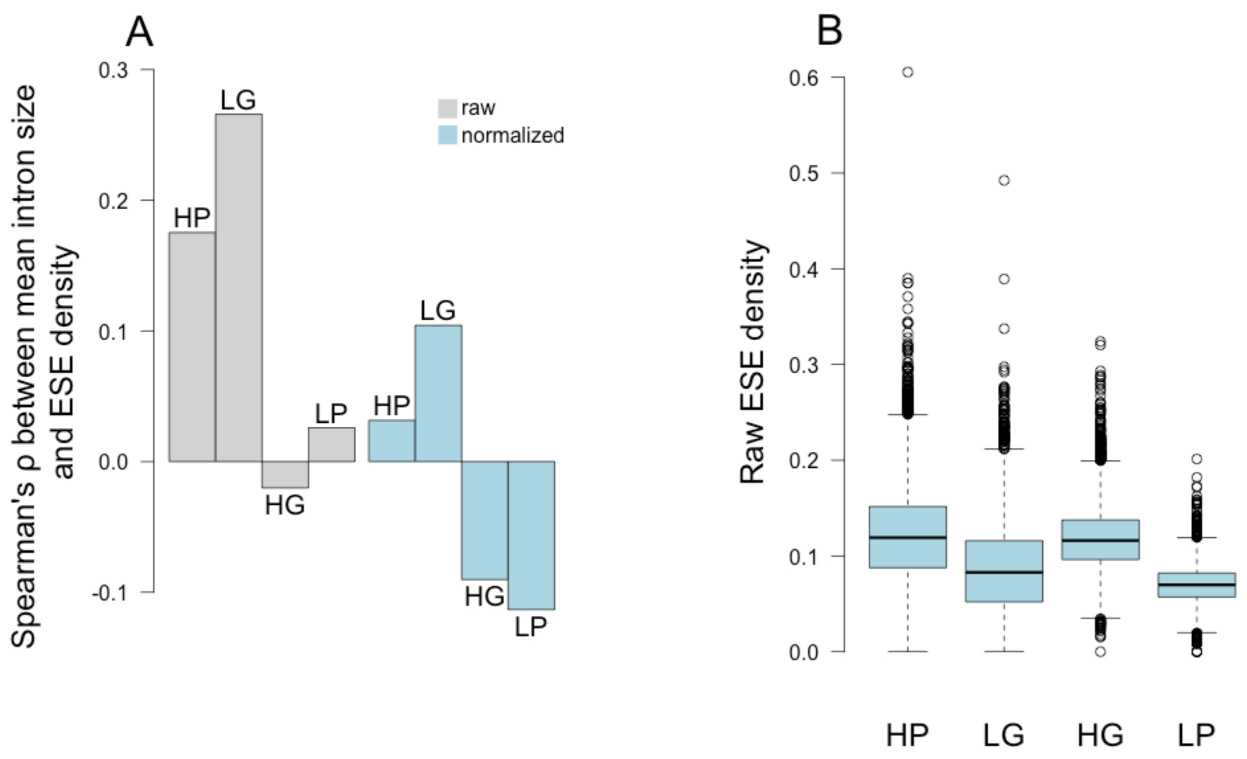

Supplementary Figure 6

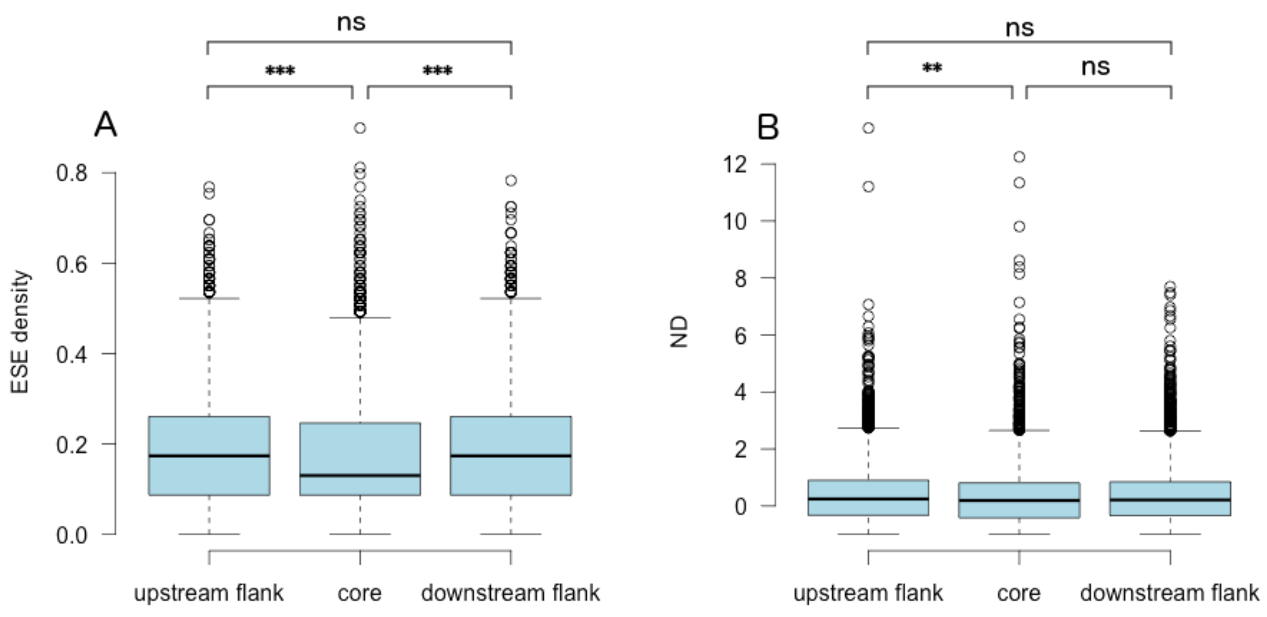

Supplementary Figure 7

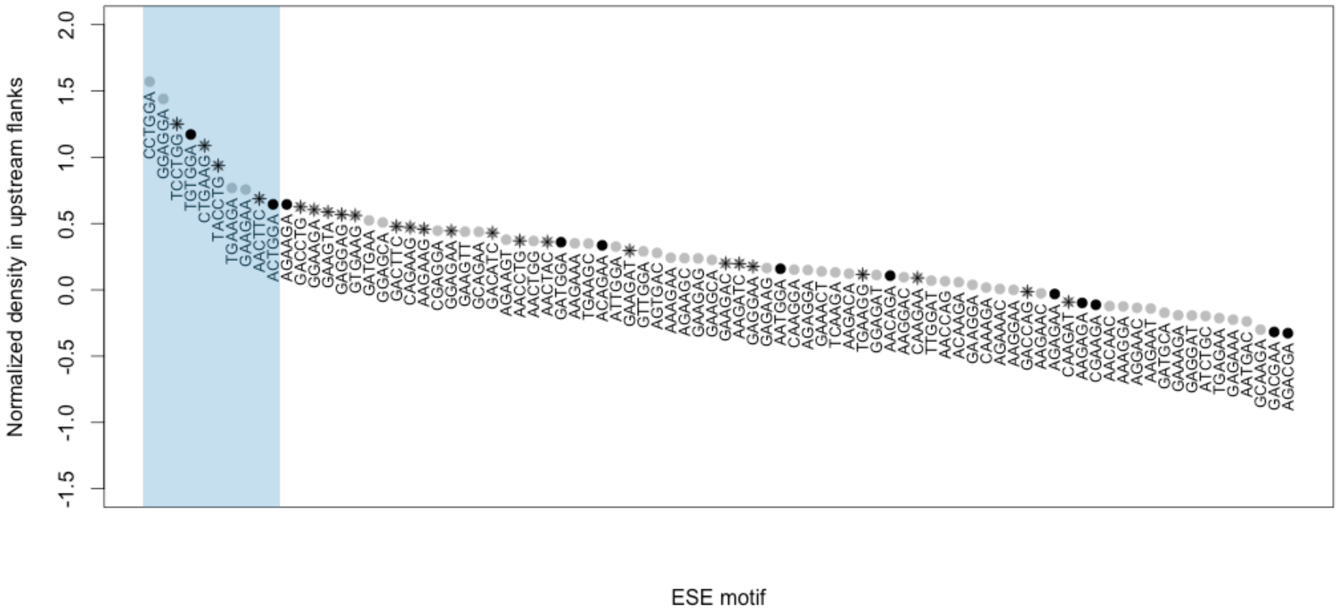

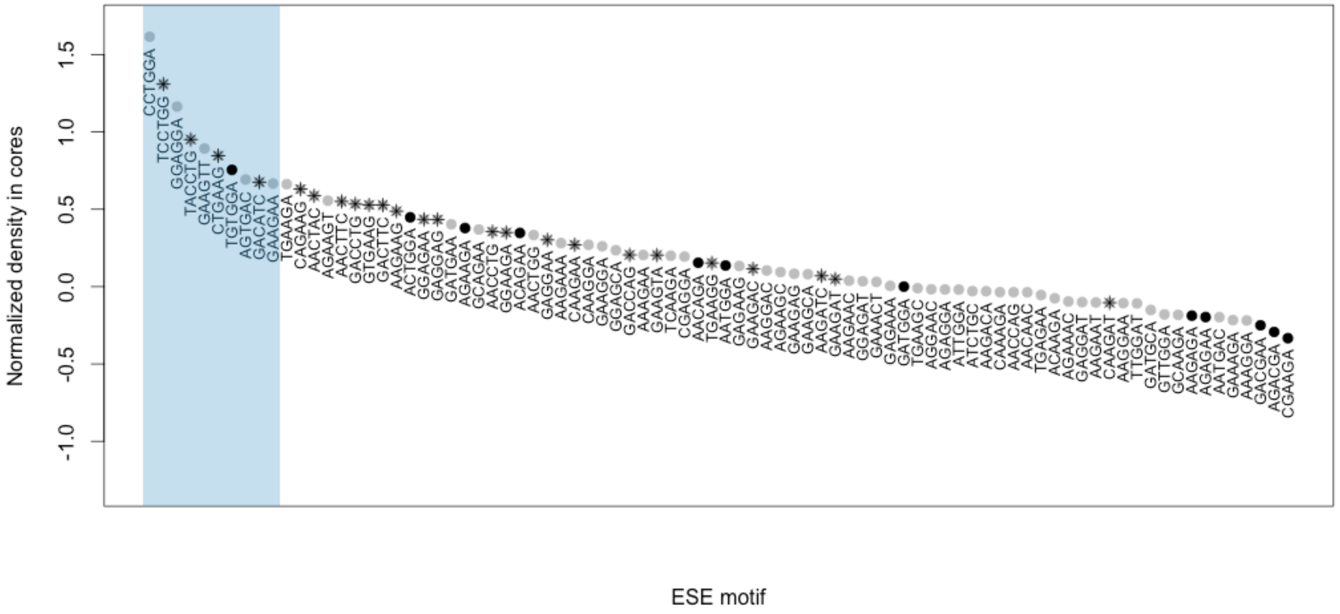

Supplementary Figure 8

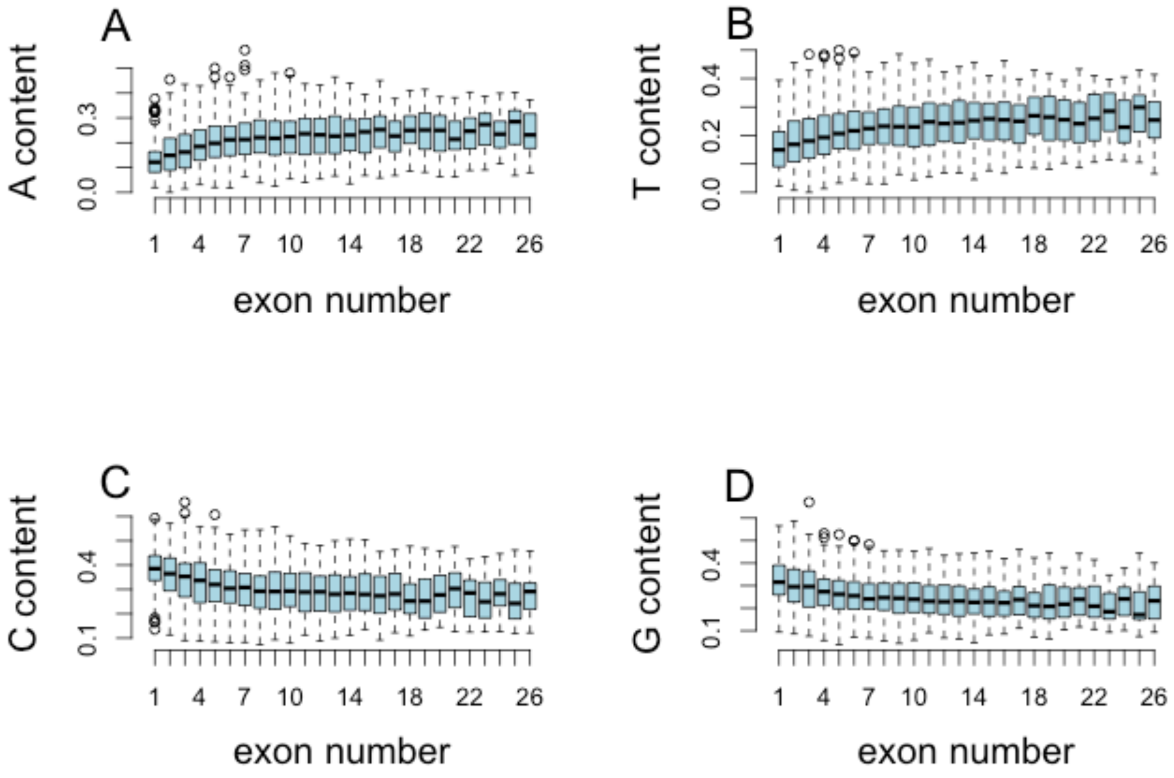

Supplementary Figure 9

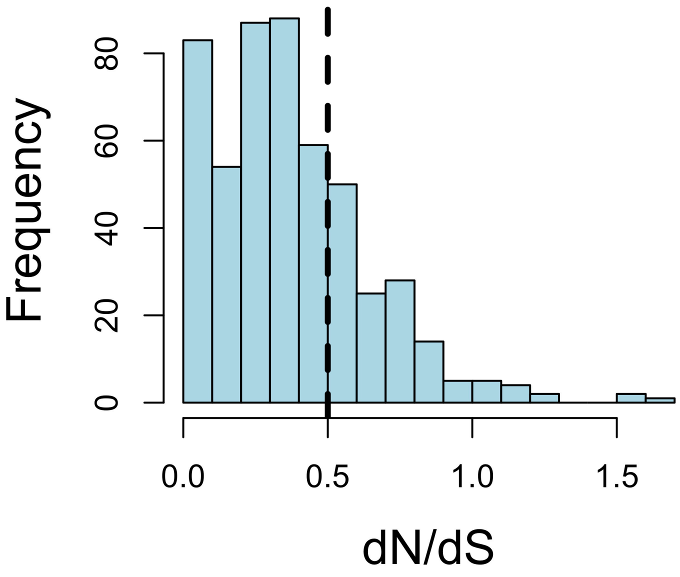

Supplementary Figure 10

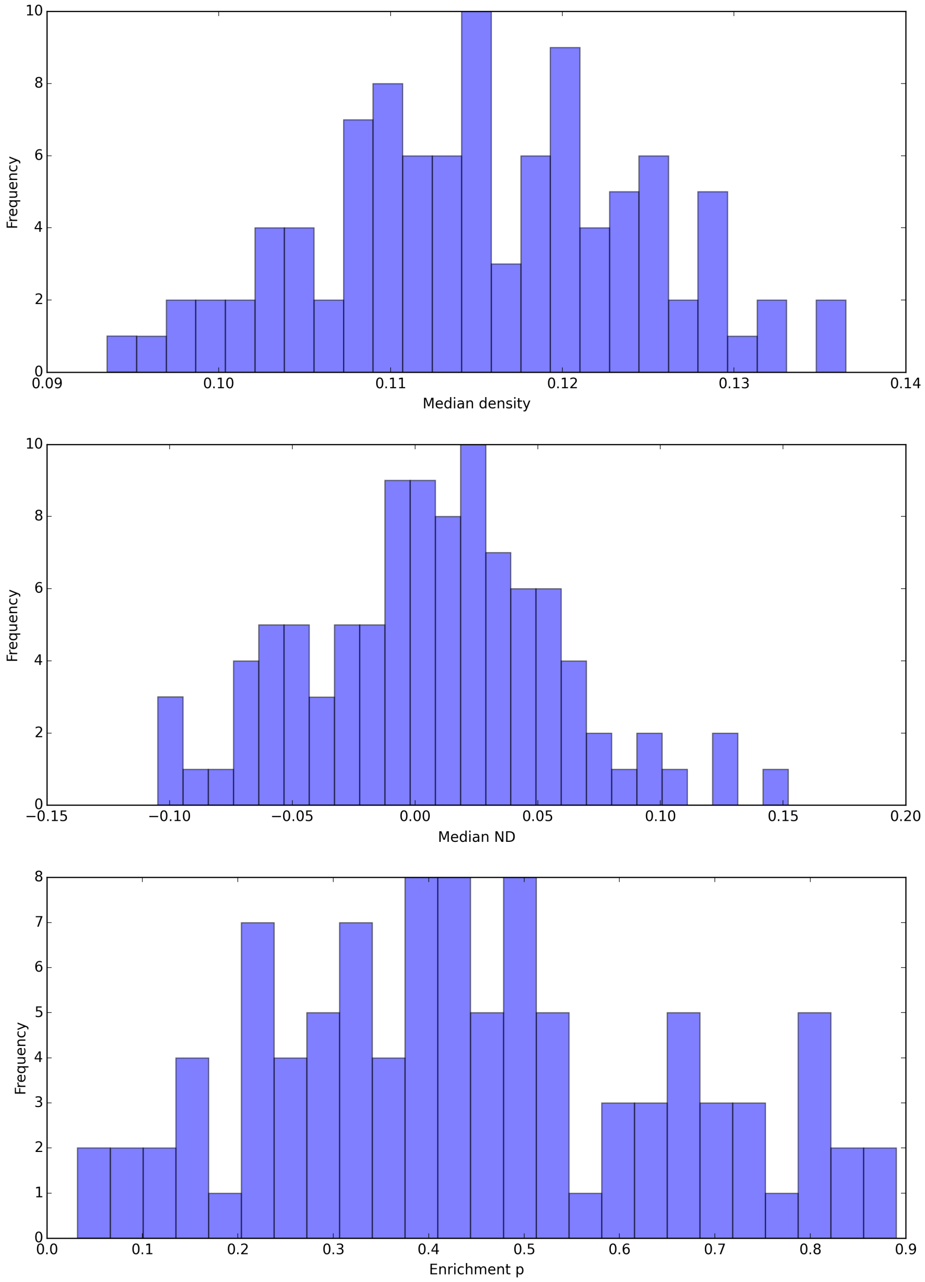

Supplementary Figure 11

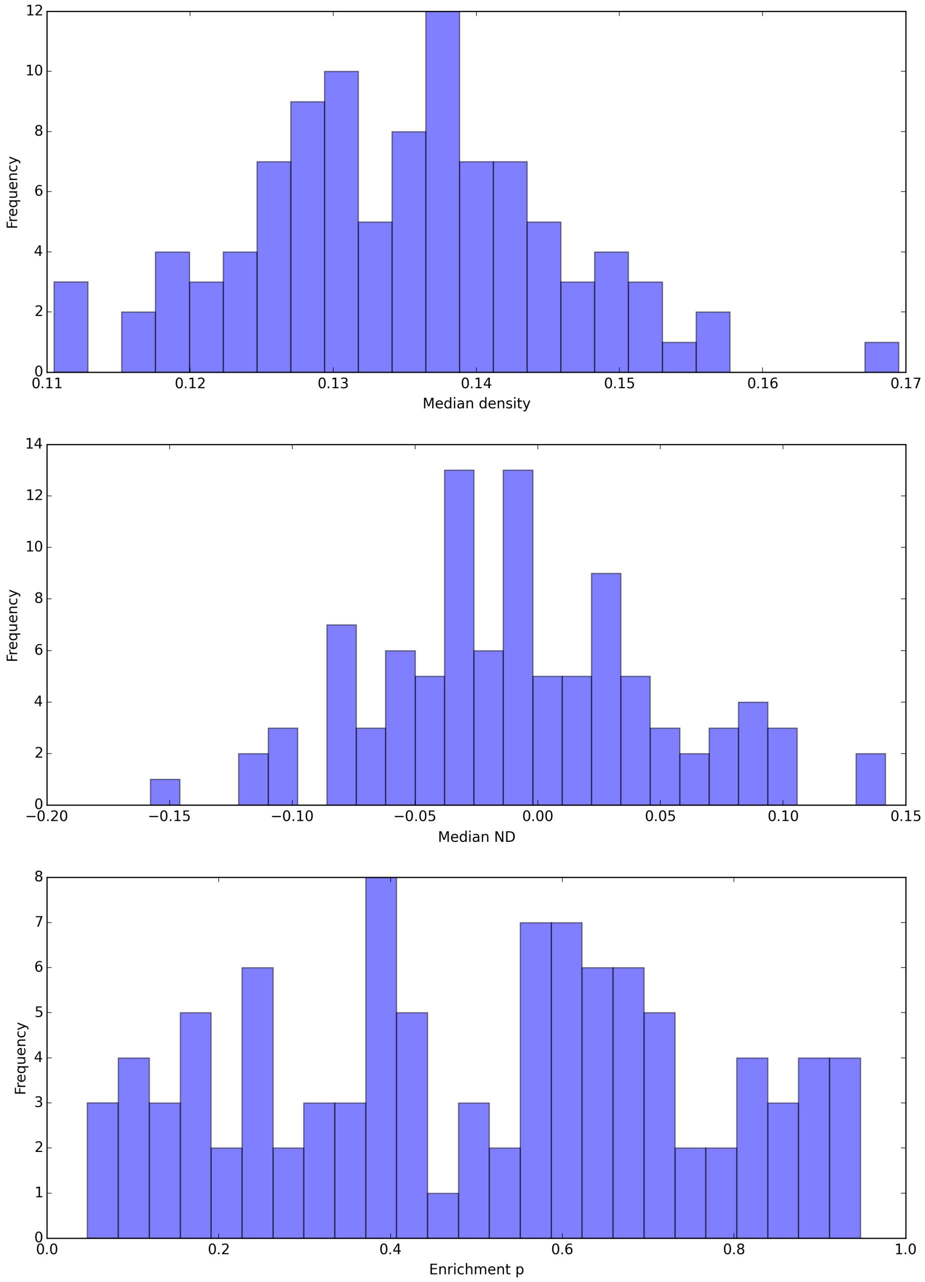

Supplementary Figure 12

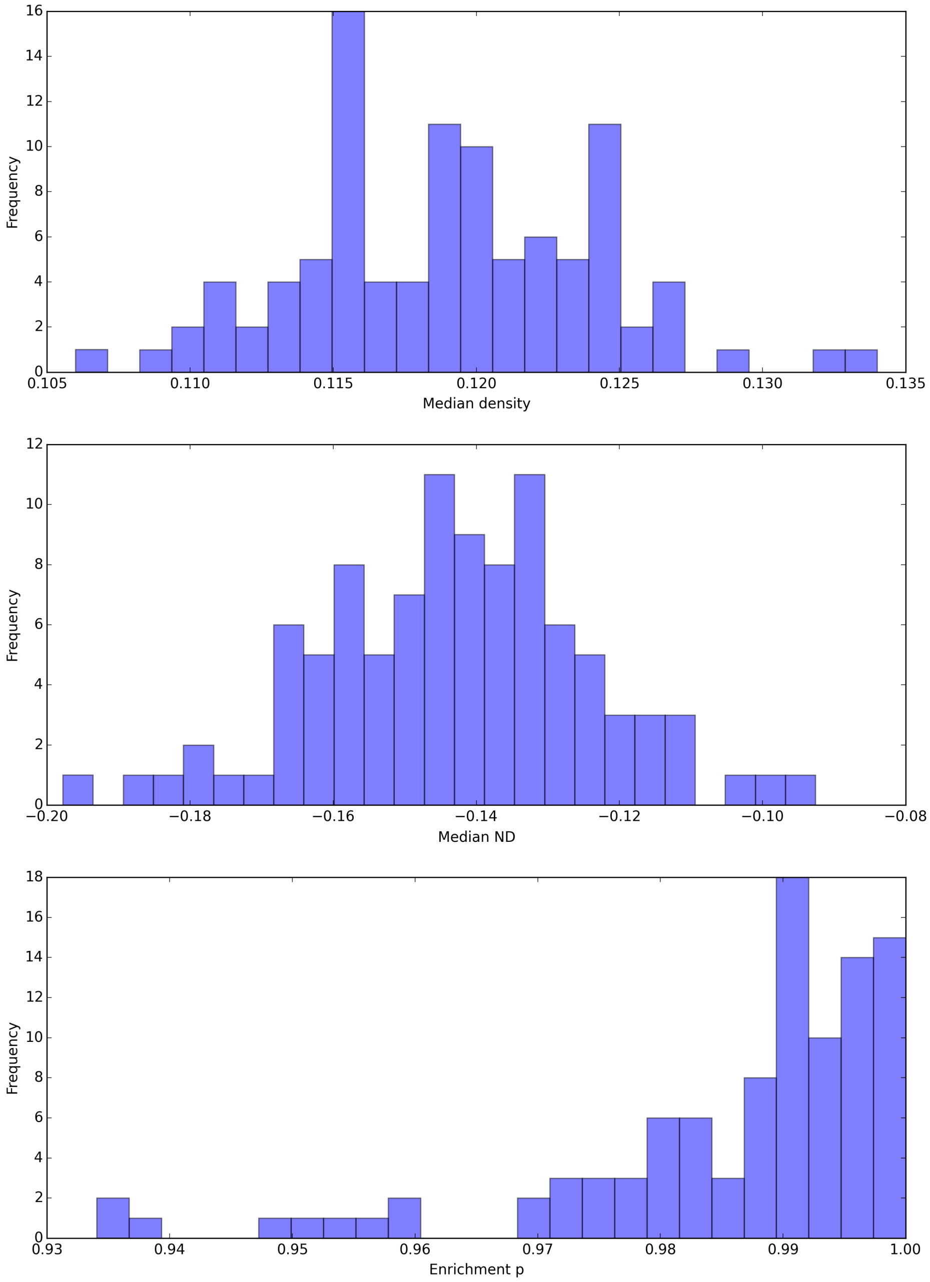

Supplementary Figure 13

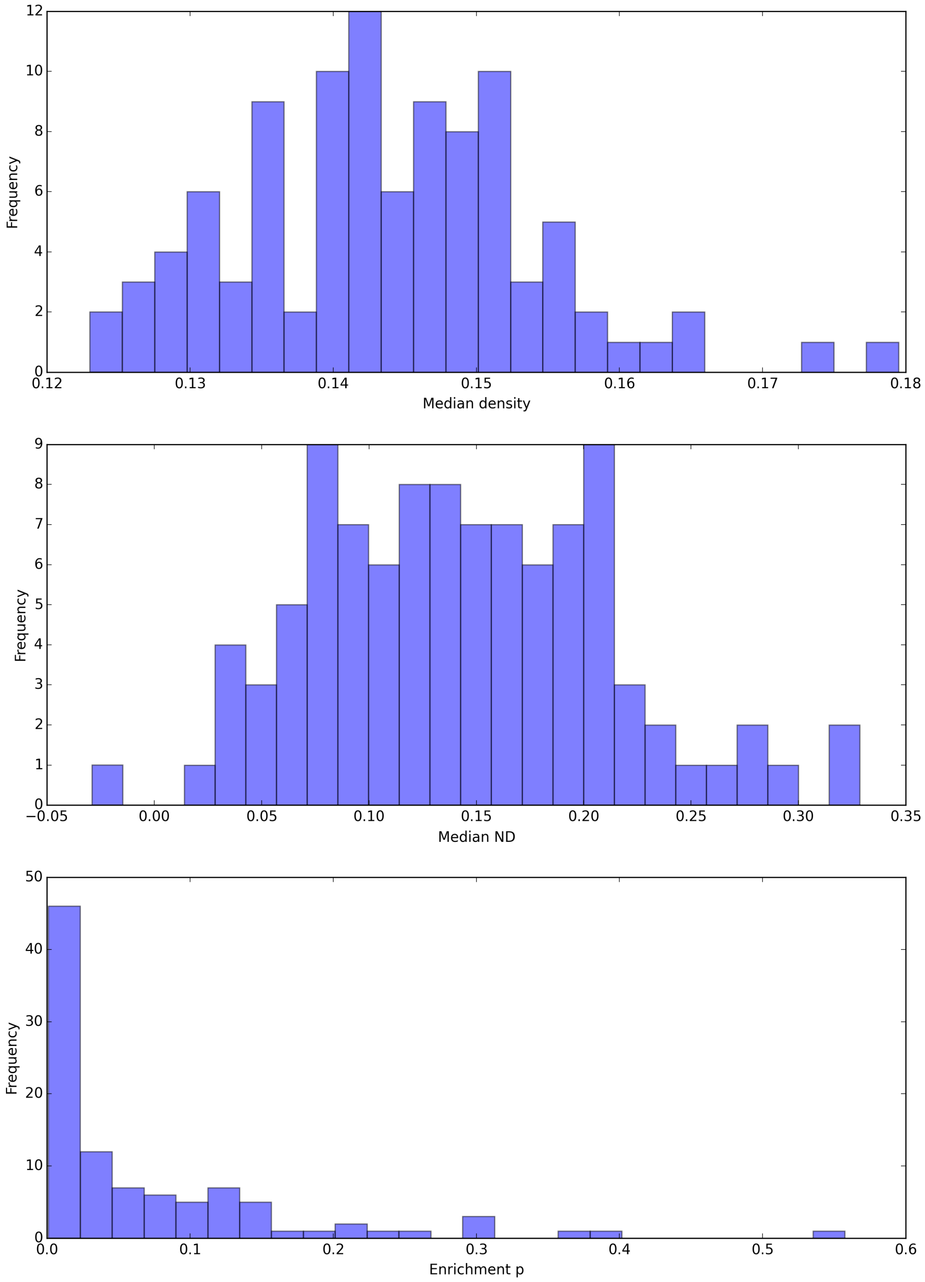

Supplementary Figure 14

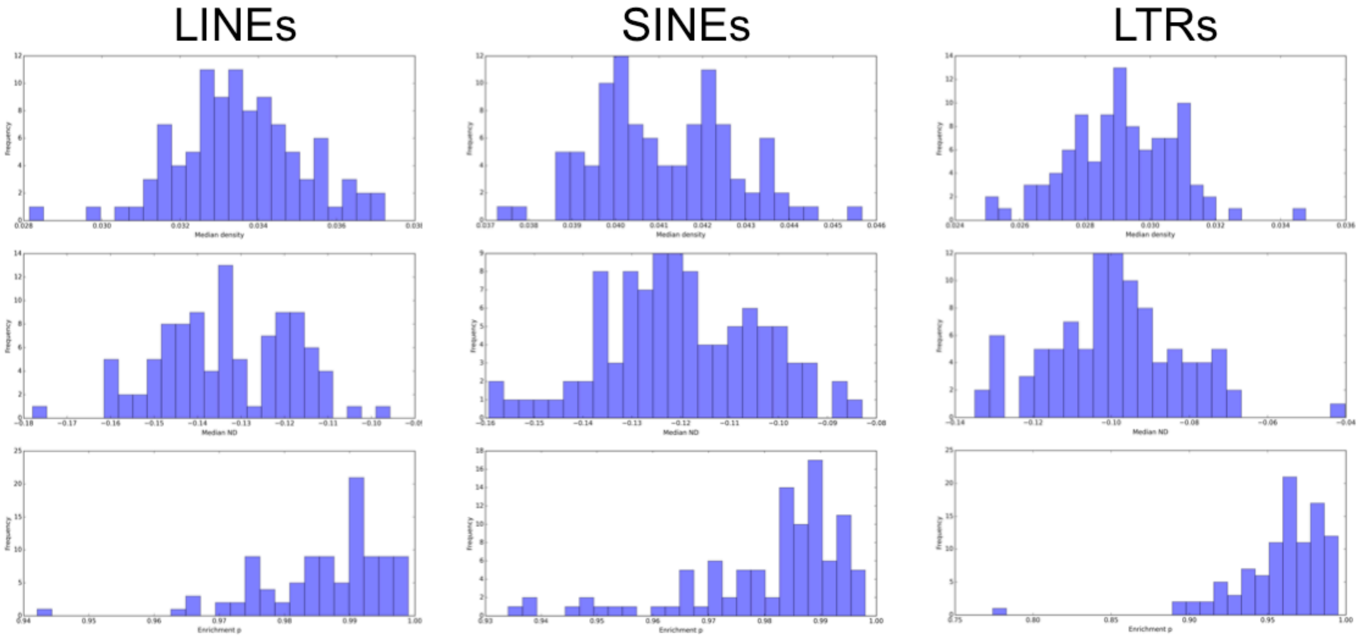

Supplementary Figure 15

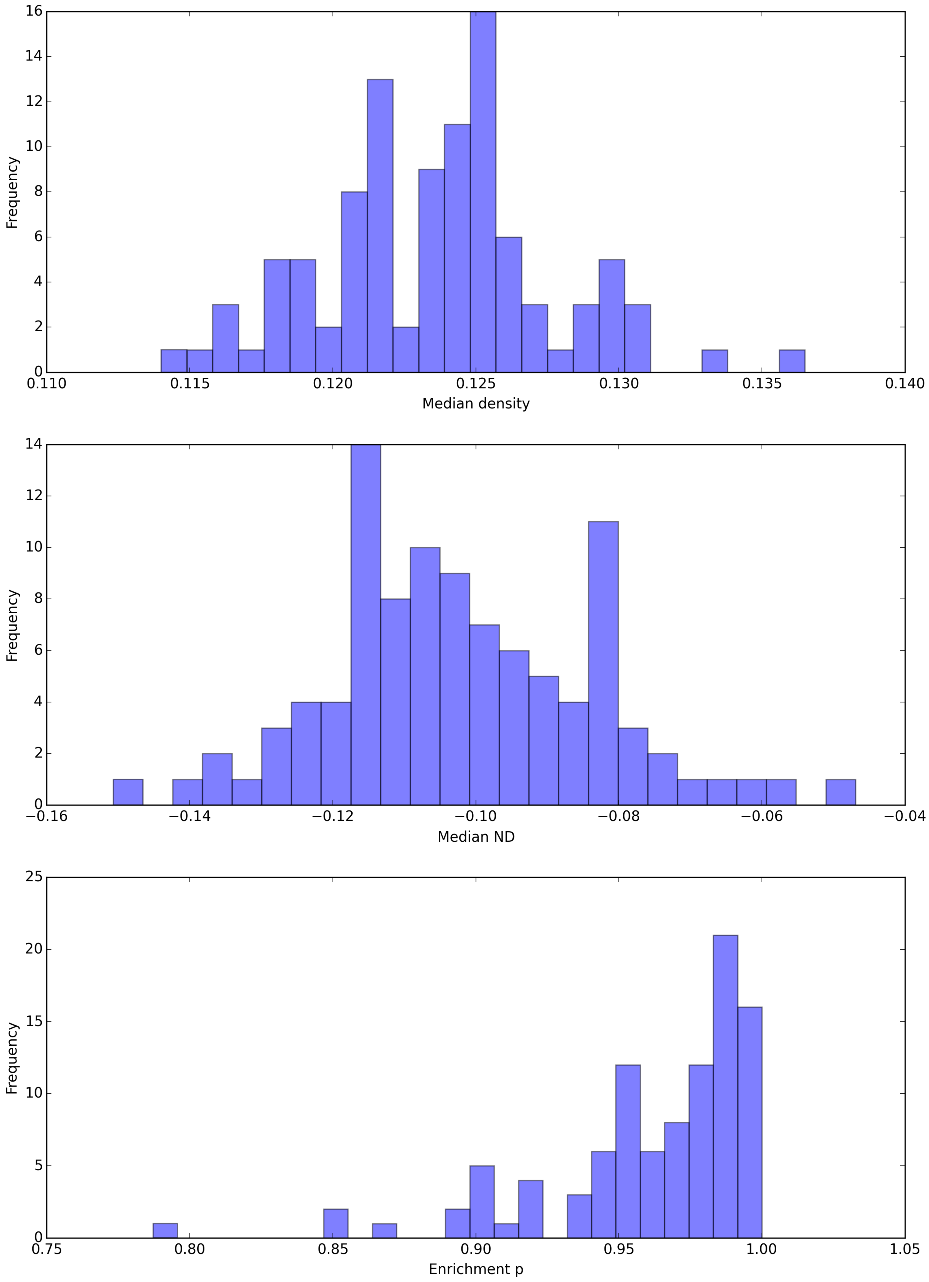

Supplementary Figure 16

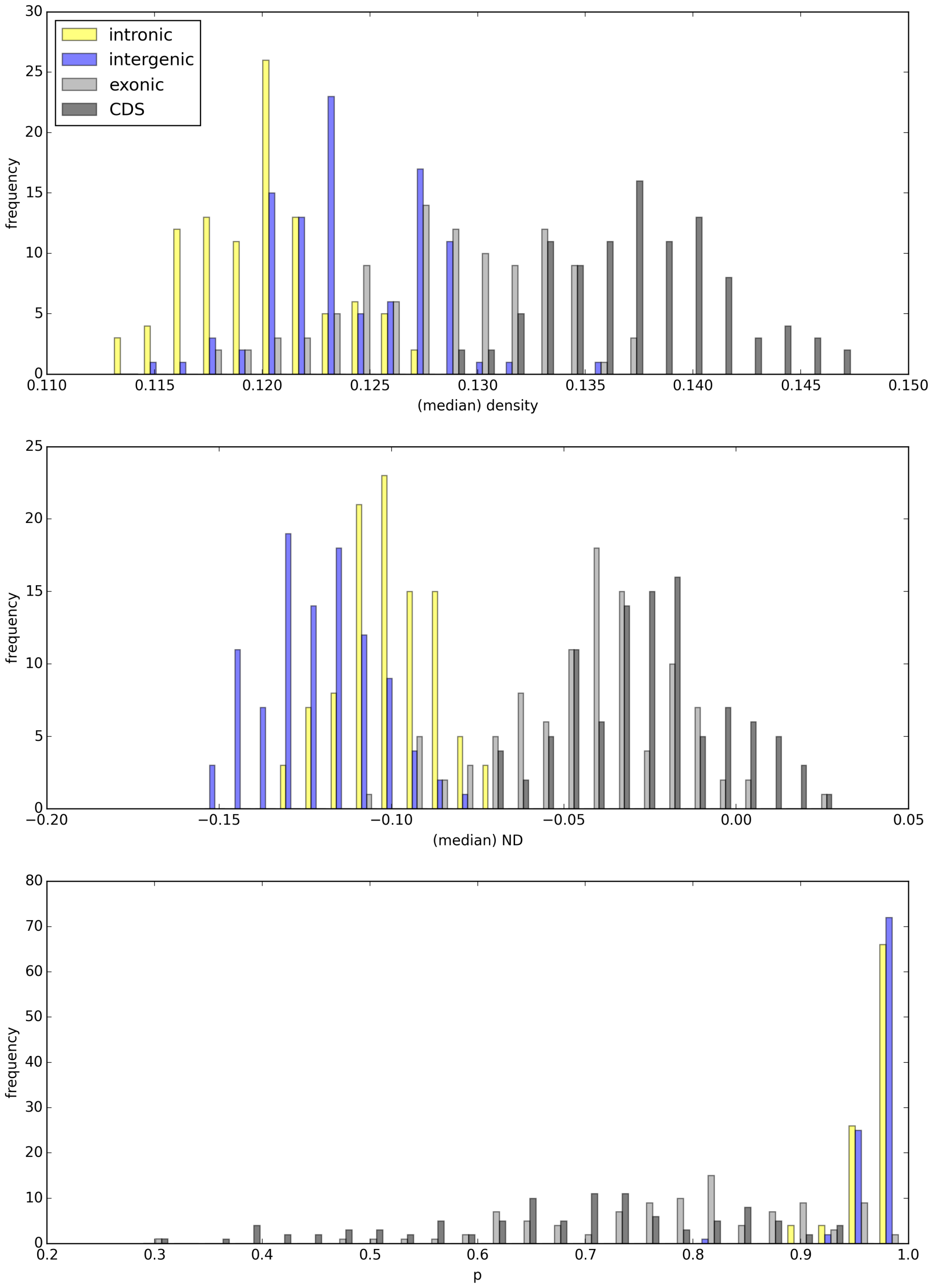

Supplementary figure legends

Supplementary Figure 1: Distribution of median densities obtained with 10,000 sets of simulated ESE motifs. The arrow shows the density of real ESEs. a) human intronless genes b) human intron-containing genes c) mouse intronless genes d) mouse intron-containing genes.

Supplementary Figure 2: Normalized ESE density (ND) in genes with different numbers of exons.

Supplementary Figure 3: ESE density (A: raw, B: normalized), rate of evolution at synonymous sites (C: raw values, D: normalized values ($= \frac{real\ d_S - simulated\ average}{simulated\ average}$)) and SNP density (E: raw values, F: normalized values ($= \frac{real\ density - simulated\ average}{simulated\ average}$)) at fourfold degenerate sites in ESEs, calculated using the broad set retrocopies, sequences not included in the broad retrocopies set and the full set of intronless genes. The asterisks in A, C and E represent empirically derived probabilities that an ESE density this high or higher, or a d_S score/SNP density this low or lower could have been obtained by chance and therefore do not pertain to any comparisons between the sets of genes. ns: $p \geq 0.05$; *: $p < 0.05$; **: $p < 0.01$; ***: $p < 0.001$. There are no error bars in the bar plots as all intronless gene ESEs were pooled to estimate the d_S scores/SNP densities reported (see *Materials and Methods*). The bar plots therefore represent single values and not distributions.

Supplementary Figure 4: a) raw ESE density b) ND and c) GC content in putative retrocopies (light blue) and their likely parents (black). The dot representing each retrocopy is linked to the dot corresponding to its presumed parent by a line.

Supplementary Figure 5: As Figure 5 a) and b) but with broad set retrocopies excluded from the dataset.

Supplementary Figure 6: raw ESE density and ND in the first 69, middle 69 and final 69 base pairs of multi-exon gene exons. *p*-values were obtained from pairwise Wilcoxon signed rank tests and were Holm-corrected for multiple testing. ns: $p \geq 0.05$; *: $p < 0.05$; **: $p < 0.01$; ***: $p < 0.001$.

Supplementary Figure 7: The top panel shows the normalized density of different ESEs in the 69 upstream-most base pairs of 4613 internal coding exons. The blue rectangle highlights the ten most frequent motifs. The shape and colour of the dot give information on the correlation between the normalized density of that motif and mean intron size bin indices. Grey circle: no significant correlation; black circle: significant positive correlation; black star: significant negative correlation. The bottom panel is identical, except that instead of the upstream-most 69 base pairs, it is 69 base pairs in the middle of the exon that are considered.

Supplementary Figure 8: a) A content, b) T content, c) C content and d) G content in genes with different numbers of exons.

Supplementary Figure 9: The distribution of dN/dS ratios for human/macaque pairs of orthologs. For human genes with several possible macaque orthologs that passed the d_S filter of 0.2, the alignment that produced the lowest ratio was considered for making the plot.

Supplementary Figure 10: The distribution of median motif densities, median ND values and enrichment *p*-values obtained by scanning a set of 100 random 1kb-long genomic regions with a different set of 84 random hexamers each time over 100 iterations.

Supplementary Figure 11: The distribution of median motif densities, median ND values and enrichment *p*-values obtained by scanning a set of 100 random 1kb-long genomic regions with a different set of 84 hexamers each time over 100 iterations, each set of motifs having been randomly sampled from the human genome mononucleotide composition.

Supplementary Figure 12: The distribution of median motif densities, median ND values and enrichment *p*-values obtained by scanning a different set of *ca.* 100 random 1kb-long genomic regions with INT3 ESEs each time over 100 iterations.

Supplementary Figure 13: When 84 random 6-basepair regions are picked from within coding regions and defined as a set of motifs, they are usually found to be enriched in random genomic regions rather than depleted. The figure shows the

distribution of median motif density, median ND and enrichment *p* values resulting from 100 such simulations where a different set of 84 motifs was picked each time and scanned for in a set of 100 random genome regions of 1kb length.

Supplementary Figure 14: The distribution of median ESE densities (INT3 set), median ND values and enrichment *p*-values obtained by scanning a set of *ca.* 1000 random 300 bp long genomic regions from either LINE, SINE or LTR elements.

Supplementary Figure 15: As Supplementary Figure 12 but with the random regions sampled from a repeatmasked genome.

Supplementary Figure 16: The distribution of median ESE densities (INT3 set), median ND values and enrichment *p*-values obtained by scanning a set of *ca.* 100 random 1kb-long genomic regions from either intergenic, intronic, exonic or coding regions. Note that the exonic regions are taken here to include coding regions, meaning that the grey and the black distribution are not independent.

Supplementary Tables

Supplementary Table 1

Base	Frequency in the INT3 set of ESEs
A	≈0.466
T	≈0.099
C	≈0.117
G	≈0.317

Supplementary Table 2

	Single-exon	Multi-exon
Median raw ESE density	≈0.131	≈0.178
Median ND	≈0.162	≈0.213
p-value	≈$9.999 * 10^{-5}$	≈$9.999 * 10^{-5}$

Supplementary Table 3

	Full dataset	Without broad set retrocopies
Median raw ESE density	≈0.131	≈0.122
Median ND	≈0.251	≈0.233
p-value	≈0.004	≈0.014

Supplementary Table 4

	Full dataset	Without broad set retrocopies	Broad set retrocopies only
Real d_S	≈0.051	≈0.052	≈0.055
Mean simulated d_S	≈0.065	≈0.064	≈0.063
Normalized d_S (*(real – simulated)/simulated*)	≈-0.208	≈-0.189	≈-0.129
p-value	≈$2.000*10^{-4}$	≈0.003	≈0.085
Sample size	157	122	50

Supplementary Table 5

	Full dataset	Without broad set retrocopies	Broad set retrocopies only
SNP density in real ESEs	≈0.044	≈0.038	≈0.057
Mean SNP-density in simulated ESEs	≈0.046	≈0.044	≈0.052
Normalized SNP density ($(\frac{real - simulated}{simulated})$)	≈-0.028	≈-0.142	≈0.102
p-value from empirical distribution	≈0.395	≈0.092	≈0.763
Sample size	157	122	50

Supplementary Table 6

	Full dataset	Without retrocopies (strict set)	Without retrocopies (broad set)
SNP density in real ESEs	≈0.044	≈0.040	≈0.038
Normalized SNP density ($\frac{real - simulated}{simulated}$)	≈-0.023	≈-0.085	≈-0.104
p-value from empirical distribution	≈0.440	≈0.204	≈0.171

Supplementary Table 7

	Intronless	Intron-containing	Total
Initial number of CDS	2253	148633	150886
After verifying exon number	1225	147670	148895
After verifying reading frame integrity and length	1142	56859	58001
After keeping only one CDS per gene	1118	19005	20123
After verifying conservation	344	10337	10681
Number of data points after clustering into paralogous families	157	5845	6002

Supplementary Table 8

	All intronless lncRNAs	conserved (3’)	conserved (CDS)	random genomic regions
median raw density	≈0.084	≈0.118	≈0.118	≈0.118
mean simulated density	≈0.101	≈0.132	≈0.129	≈0.138
median ND	≈-0.15	≈-0.124	≈-0.133	≈-0.138
enrichment *p*-value	≈0.991	≈0.941	≈0.864	≈0.996
sample size	157 datapoints (262 genes)	69 datapoints (102 genes)	41 datapoints (70 genes)	949 datapoints (949 genes - no clustering into families was performed)

Supplementary table legends

Supplementary Table 1: Base composition of the INT3 set of ESEs used in this study (see *Materials and Methods* for more details on the set of motifs)

Supplementary Table 2: raw and normalized ESE density, using the empirical distribution derived from predicting hits to ESEs in 10,000 simulated versions of the single-exon/multi-exon dataset where the codons had been shuffled within each CDS (see *Methods* for further details).

Supplementary Table 3: raw and normalized ESE density with and without the putative retrocopies, using only 1000 simulants to calculate ND and the *p*-value.

Supplementary Table 4: Rate of evolution at synonymous sites in ESEs in the full set of intronless genes and in the two retrocopyless sets.

Supplementary Table 5: SNP density at fourfold degenerate sites in ESEs in the full set of intronless genes, the broad set retrocopies and in other intronless sequences.

Supplementary Table 6: SNP density at fourfold degenerate sites in ESEs in the full set of intronless genes, the broad set retrocopies and in other intronless sequences, using only 1000 simulants to calculate ND and the *p*-value.

Supplementary Table 7: number of CDS remaining in the dataset after the various filtering steps.

Supplementary Table 8: Density of ESE hexamers and associated statistics, calculated in putative intronless lncRNAs or randomly selected 1kb regions from anywhere in the genome.

References

Grosso, A.R., Gomes, A.Q., Barbosa-Morais, N.L., Caldeira, S., Thorne, N.P., Grech, G., von Lindern, M., and Carmo-Fonseca, M. (2008). Tissue-specific splicing factor gene expression signatures. Nucleic Acids Res *36*, 4823-4832.

Han, J., Ding, J.H., Byeon, C.W., Kim, J.H., Hertel, K.J., Jeong, S., and Fu, X.D. (2011). SR proteins induce alternative exon skipping through their activities on the flanking constitutive exons. Mol Cell Biol *31*, 793-802.

Hanamura, A., Caceres, J.F., Mayeda, A., Franza, B.R.J., and Krainer, A.R. (1998). Regulated tissue-specific expression of antagonistic pre-mRNA splicing factors. RNA *4*, 430-444.

Hangauer, M.J., Vaughn, I.W., and McManus, M.T. (2013). Pervasive transcription of the human genome produces thousands of previously unidentified long intergenic noncoding RNAs. PLoS Genet *9*, e1003569.

Lynch, M., and Walsh, B. (2007). The origins of genome architecture, Vol 98 (Sinauer Associates Sunderland).

Schüler, A., Ghanbarian, A.T., and Hurst, L.D. (2014). Purifying Selection on Splice-Related Motifs, Not Expression Level nor RNA Folding, Explains Nearly All Constraint on Human lincRNAs. Molecular biology and evolution, msu249.

Siepel, A., Bejerano, G., Pedersen, J.S., Hinrichs, A.S., Hou, M., Rosenbloom, K., Clawson, H., Spieth, J., Hillier, L.W., Richards, S.*, et al.* (2005). Evolutionarily conserved elements in vertebrate, insect, worm, and yeast genomes. Genome Res *15*, 1034-1050.

Smit, A.F.A., Hubley, R., and Green, P. (2013-2015). RepeatMasker.
